# Supplementary material for: Stingless bee honey: Nutritional, physicochemical, phytochemical and antibacterial validation properties against wound bacterial isolates
Source: PLoS One. 2024 May 14;19(5):e0301201. doi: 10.1371/journal.pone.0301201 (PMC11093306; doi:10.1371/journal.pone.0301201)
Supplement: S1 Fig — (PDF) [file pone.0301201.s001.pdf]

S1 Fig. Agarose gel (1%) electrophoresis. Figure 1

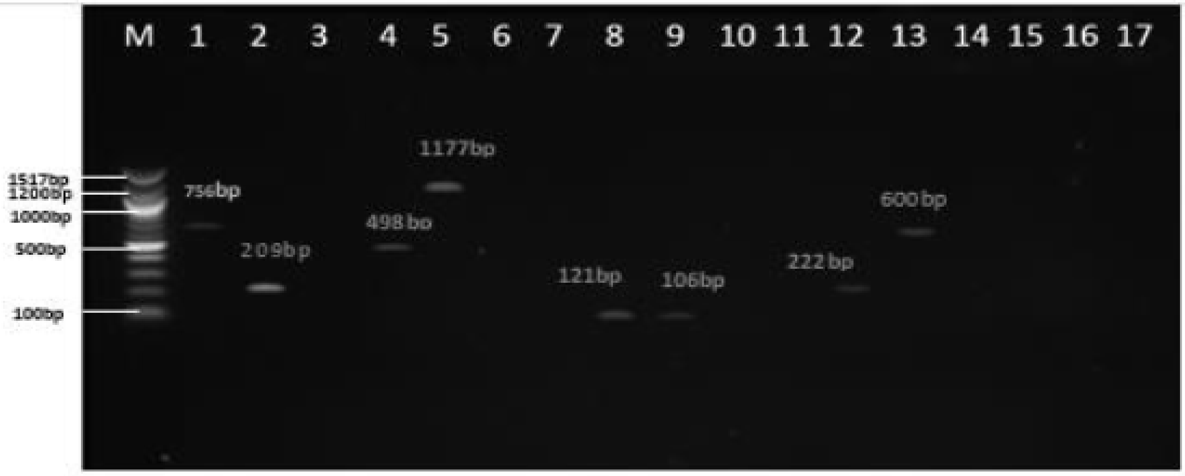

Figure 1: Agarose gel (1%) electrophoresis showing the typical amplicons of the virulence genes products of the bacterial isolates *Staphylococcus aureus (*Lane 1 and 2), *Escherichia coli (*lane 4 and 5), *Klebsiella pneumoniae (*Lane 8 and 9) and *Pseudomonas aeruginosa* (Lane 12 and 13). Lanes 6 and 10 were negative for the genes and hence did not indicate any amplicons while lanes 3, 7 and 11 were set as negative control. M is a ladder (standard) containing a mixture of DNA fragments of predetermined sizes that can be compared against the unknown DNA samples.
