# Supplementary material for: Stingless bee honey: Nutritional, physicochemical, phytochemical and antibacterial validation properties against wound bacterial isolates
Source: PLoS One. 2024 May 14;19(5):e0301201. doi: 10.1371/journal.pone.0301201 (PMC11093306; doi:10.1371/journal.pone.0301201)
Supplement: S2 Fig — (PDF) [file pone.0301201.s002.pdf]

S2 Fig. Physicochemical nutritive properties of stingless honey. Figure 2

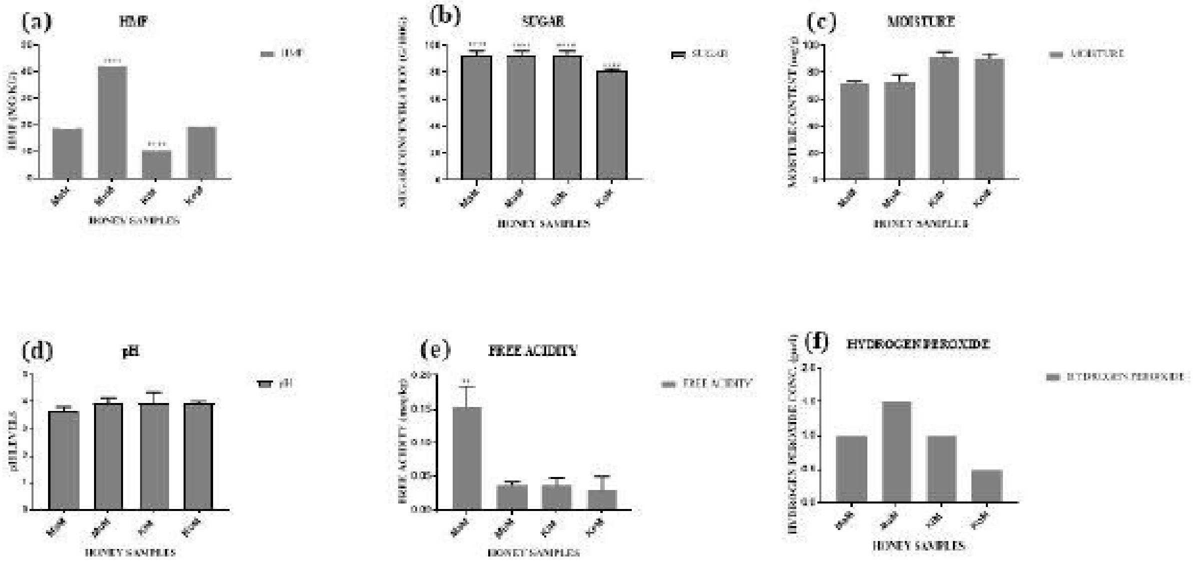

Figure 2: Physicochemical and nutritive properties of stingless bee honey; Hydromethylfurfural – HMF (a), Sugar (b), Moisture (c), pH (d), Free acidity (e) and Hydrogen peroxide (f). The values are represented in mean ± SD as error bars represent Standard deviation (SD). Significant values ($P<0.05$) compared to a standard are represented by stars on the bars (*$P< 0.05$, **$P<0.01$, ***$P<0.001$ and **** $P<0.0001$). (**KEY**: **MaM** – Maoi Meliponin, **MuM** – Mukutani Meliponin, **KiM** – Kibigor Meliponin, **KoM**- Koriema Meliponin).
