# Supplementary material for: Stingless bee honey: Nutritional, physicochemical, phytochemical and antibacterial validation properties against wound bacterial isolates
Source: PLoS One. 2024 May 14;19(5):e0301201. doi: 10.1371/journal.pone.0301201 (PMC11093306; doi:10.1371/journal.pone.0301201)
Supplement: S4 Fig — (PDF) [file pone.0301201.s004.pdf]

S4 Fig. Physicochemical nutritive properties of stingless honey. Figure 4

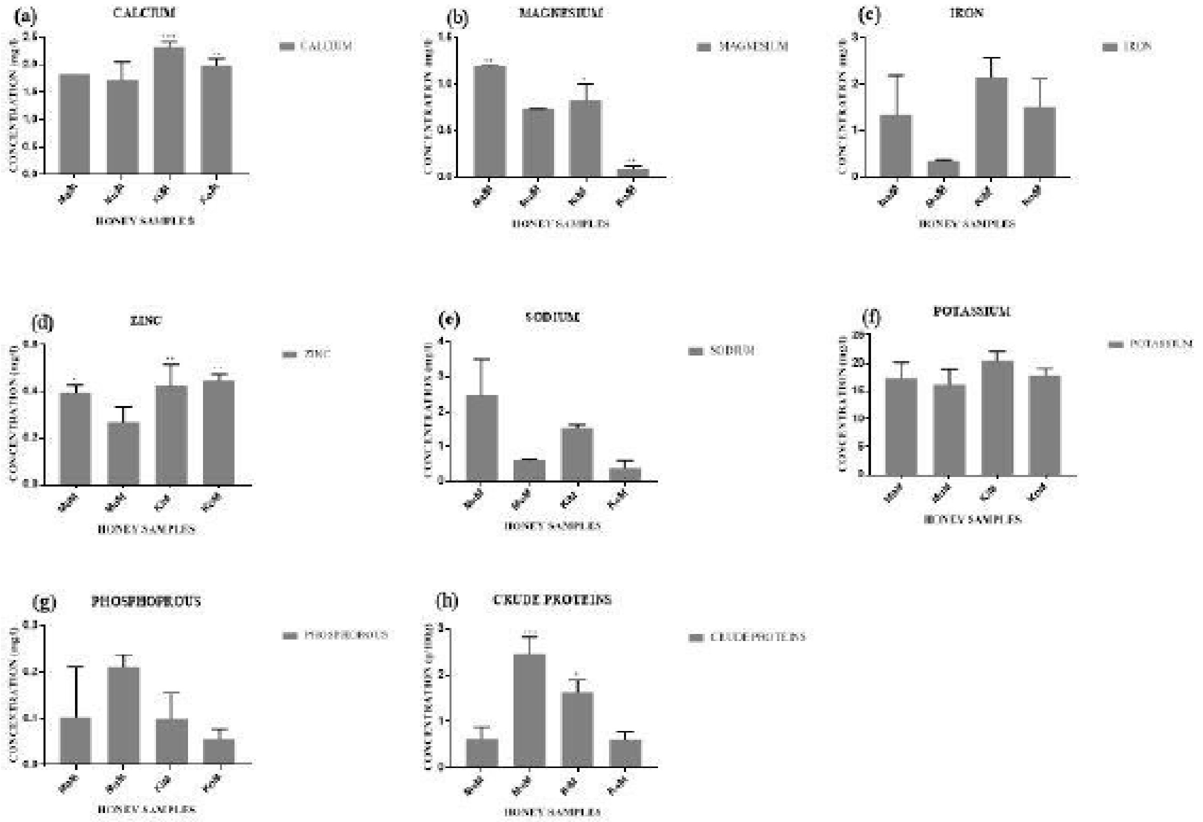

Figure 4: Physicochemical and nutritive properties of stingless bee honey; Calcium – ca (a), Magnesium – mg (b), Iron- fe (c), Zinc – zn (d) Sodium – Na (e), Potassium - K (f), Phosphorous - P (g) and Crude proteins (h). The values are represented in mean ± SD as error bars represent Standard deviation (SD). Significant values ($P<0.05$) compared to a standard are represented by stars on the bars (*$P< 0.05$,**$P<0.01$, ***$P<0.001$ and **** $P<0.0001$) (**KEY: MaM** – Maoi Meliponin, **MuM** – Mukutani Meliponin, **KiM** – Kibigor Meliponin, **KoM**- Koriema Meliponin).
