# Supplementary material for: Stingless bee honey: Nutritional, physicochemical, phytochemical and antibacterial validation properties against wound bacterial isolates
Source: PLoS One. 2024 May 14;19(5):e0301201. doi: 10.1371/journal.pone.0301201 (PMC11093306; doi:10.1371/journal.pone.0301201)
Supplement: S5 Fig — (PDF) [file pone.0301201.s005.pdf]

S5 Fig. Phytochemical properties of stingless honey. Figure 5.

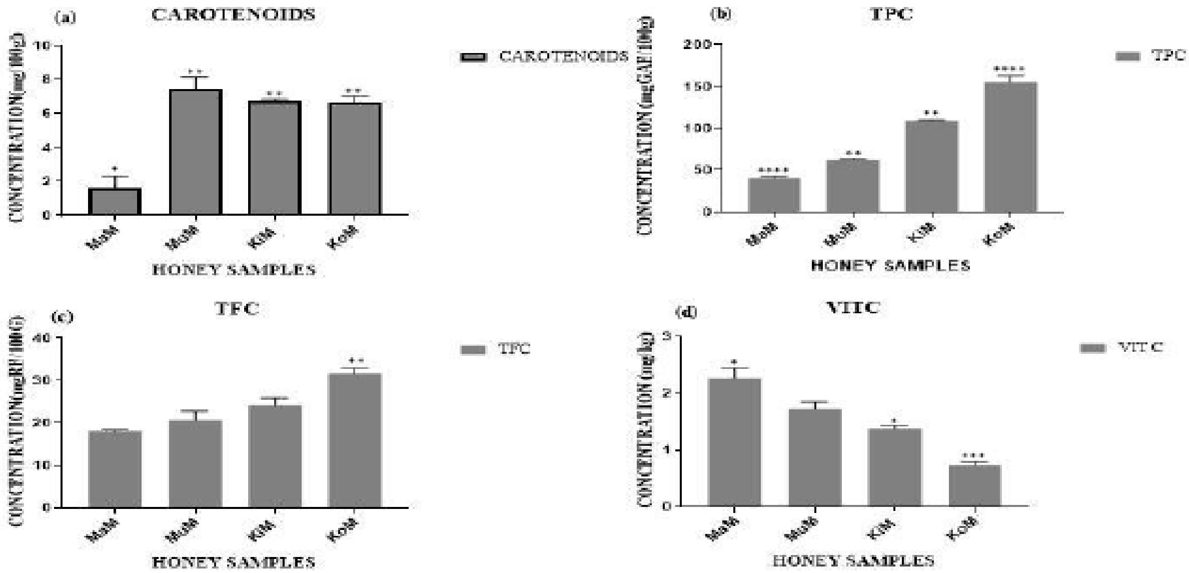

Figure 5: Phytochemical properties of stingless bee honey; Total flavonoid content – TFC (a), Total phenolic content – TPC (b), Carotenoids (c) and Vitamin C (d). The values are represented in mean ± SD as error bars represent Standard deviation (SD). Significant values ($P<0.05$) compared to a standard are represented by stars on the bars (*$P< 0.05$,**$P<0.01$, ***$P<0.001$ and **** $P<0.0001$) (**KEY: MaM** – Maoi Meliponin, **MuM** – Mukutani Meliponin, **KiM** – Kibigor Meliponin, **KoM**- Koriema Meliponin).
