# Supplementary material for: Stingless bee honey: Nutritional, physicochemical, phytochemical and antibacterial validation properties against wound bacterial isolates
Source: PLoS One. 2024 May 14;19(5):e0301201. doi: 10.1371/journal.pone.0301201 (PMC11093306; doi:10.1371/journal.pone.0301201)
Supplement: S6 Fig — (PDF) [file pone.0301201.s006.pdf]

S6 Fig. Antibacterial activity of commonly used antibiotics. Figure 6.

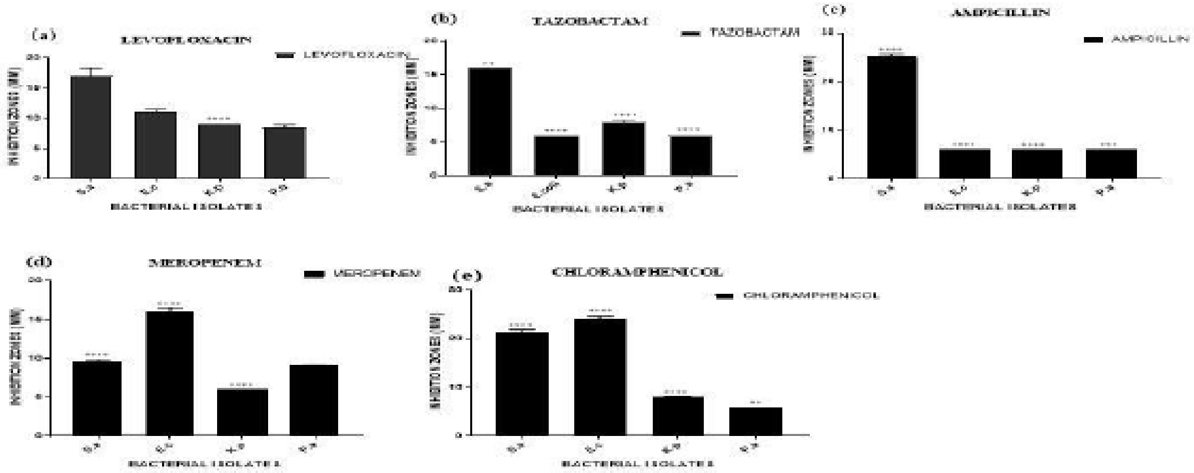

Figure 6: Antibacterial activity of commonly used antibiotics against bacterial isolates from burns and cutaneous wounds. Levofloxacin (a), Tazobactum (b), Ampicillin (c) Meropenem (d), Gentamicin (e) and Chloramphenicol (f). The values are represented in mean ± SD as error bars represent Standard deviation (SD). Significant values ($P<0.05$) compared to the bacteria control isolates, are represented by stars on the bars (*$P< 0.05$, **$P<0.01$, ***$P<0.001$ and **** $P<0.0001$. (**KEY**: **S. a**- *Staphylococcus aureus*, **E. c.** - *Escherichia coli*, **K. p.** - *Klebsiella pneumoniae* and **P.a.** - *Pseudomonas aeruginosa*).
