# Supplementary material for: Stingless bee honey: Nutritional, physicochemical, phytochemical and antibacterial validation properties against wound bacterial isolates
Source: PLoS One. 2024 May 14;19(5):e0301201. doi: 10.1371/journal.pone.0301201 (PMC11093306; doi:10.1371/journal.pone.0301201)
Supplement: S1 Table — (PDF) [file pone.0301201.s007.pdf]

**S1 Table. Cultural proportion of pure bacterial isolates from infected cutaneous wounds.**

**Table 1**

| Type of swabs | No. collected | No. infected | No. of pure isolates | Frequency (%) |
|---|---|---|---|---|
| Burn | 6 | 4 | 6 | 14.7 |
| Wound | 28 | 24 | 37 | 82.4 |
| **Total** | 34 | 28 | 43 | 97.1 |
