# Supplementary material for: Stingless bee honey: Nutritional, physicochemical, phytochemical and antibacterial validation properties against wound bacterial isolates
Source: PLoS One. 2024 May 14;19(5):e0301201. doi: 10.1371/journal.pone.0301201 (PMC11093306; doi:10.1371/journal.pone.0301201)
Supplement: S2 Table — (PDF) [file pone.0301201.s008.pdf]

S2 Table. Proportion of Gram staining reactions of bacterial isolates from the collected wound swabs. Table 2.

Bacterial isolates	Gram reaction	Morphology	Swabs	Frequency (%)
Gram stain 1	Gram Positive	Cocci	10	29.4
Gram stain 2	Gram Negative	Rods (Single/pair)	12	35.3
Gram stain 3	Gram Negative	Rods (Plump/short chains	4	11.8
Gram stain 4	Gram Negative	Rods (Slimmer)	3	8
Gram stain 5	Nil		5	14.7
**Total**			34	91.2
