# Supplementary material for: Stingless bee honey: Nutritional, physicochemical, phytochemical and antibacterial validation properties against wound bacterial isolates
Source: PLoS One. 2024 May 14;19(5):e0301201. doi: 10.1371/journal.pone.0301201 (PMC11093306; doi:10.1371/journal.pone.0301201)
Supplement: S3 Table — (PDF) [file pone.0301201.s009.pdf]

S3 Table. Biochemical tests. Table 3.

		Gram reaction			
**Biochemical test**		**Gram-negative n=3 (75%)**		**Gram -positive n=1(25%)**	
Coagulase				+ (35%)	- 0%)
TSI	Butt/slope	Acid/Acid	Acid/ Acid	Alkaline/Alkaline	Alkaline/ Alkaline
		+ (37.2%)	+	- (62.8%)	-
	Gas	+ (37.2%)	+	- (62.8%)	-
	H2S	-	-	-	-
MethylRed		+ (13.95%)	- (23.26%)		
Oxidase		-	-	+ (27.91%)	- (13.95%)
Indole					
Voges prosker		NT	NT	NT	NT
Citrate		NT	NT	NT	NT
Urease		NT	NT	NT	NT
Possible pathogen		*E.coli* (13.95%)	*K. pneumoniae* (23.26%)	*P. aeruginosa* (27.91%)	*S. aureus* (35%)
Key	NT	Not done			
