# Supplementary material for: Stingless bee honey: Nutritional, physicochemical, phytochemical and antibacterial validation properties against wound bacterial isolates
Source: PLoS One. 2024 May 14;19(5):e0301201. doi: 10.1371/journal.pone.0301201 (PMC11093306; doi:10.1371/journal.pone.0301201)
Supplement: S4 Table — (PDF) [file pone.0301201.s010.pdf]

S4 Table. Mean inhibition of the stingless bee honey dilutions. Table 4.

		Stingless honey samples (*Meliponin*) zones of inhibition (mm)			
**Isolates**	**Concentration($x10^4$µg/disc)**	MuM	MaM	KiM	KoM
S. aureus	10	6.4 ± 0.00	6.1 ± 0.00	6.0 ± 0.00	6.0 ± 0.00
	20	6.4 ± 0.00	6.2 ± 0.00	6.0 ± 0.00	6.0 ± 0.00
	50	12.8 ± 2.17	20.08 ± 0.67	14.62 ± 0.33	21.73 ± 0.37
	75	15.29 ± 0.31	22.9 ± 0.00	17.56 ± 0.53	25.22 ± 0.49
E. coli	10	6.2 ± 0.00	6.3 ± 0.00	6.0 ± 0.00	6.1 ± 0.00
	20	6.4 ± 0.00	6.1 ± 0.00	6.0 ± 0.00	6.2 ± 0.00
	50	9.00 ± 0.37	7.11 ± 0.19	12.55 ± 0.22	7.59 ± 0.19
	75	12.48 ± 0.52	10.89 ± 0.00	15.87 ± 0.37	10.48 ± 0.00
K. Pneumoniae	10	6.1 ± 00	6.3 ± 0.00	6.0 ± 0.00	6.2 ± 0.00
	20	6.1 ± 0.00	6.0 ± 0.00	6.0 ± 0.00	6.6 ± 0.00
	50	13.48 ± 0.27	19.65 ± 0.38	15.43± 0.46	14.88 ± 0.22
	75	18.67 ± 0.39	22.96 ± 0.00	18.86 ± 0.69	17.14 ± 0.16
P. aeruginosa	10	6.3 ± 0.00	6.0 ± 0.00	6.0 ± 0.00	6.0 ± 0.00
	20	6.3 ± 0.00	6.1 ± 0.00	6.0 ± 0.00	6.6 ± 0.00
	50	16.28 ± 0.06	15.45 ± 0.35	17.89 ± 0.03	13.52 ± 0.41
	75	19.75 ± 0.12	17.19 ± 0.03	21.63 ± 0.08	18.48 ± 0.35

KEY: **MuM:** Mukutani Meliponin, **MuA**: Mukutani Apis, **MaM**:Maoi Meliponin, **KiM**: Kibingor Meliponin, **KiA**: Kibingor Apis, **KoM**: Koriema Meliponin & **KoA**: Koriema Apis
