# Supplementary material for: Stingless bee honey: Nutritional, physicochemical, phytochemical and antibacterial validation properties against wound bacterial isolates
Source: PLoS One. 2024 May 14;19(5):e0301201. doi: 10.1371/journal.pone.0301201 (PMC11093306; doi:10.1371/journal.pone.0301201)
Supplement: S5 Table — (PDF) [file pone.0301201.s011.pdf]

S5 Table. Average inhibition of the stingless honey samples. Table 5.

Isolates/ PC	Stingless bee samples (*Meliponin*) inhibition zones (mm)
S. aureus	18.79 ± 4.49
ATCC 25923 *(S.aureus)*	16.21 ± 2.64
E. coli	10.76 ± 2.46
ATCC 25922 *(E.coli)*	11.00 ± 2.44
K. pneumonia	17.55 ± 2.48
ATCC 27736(*K.pneumoniae*)	17.00 ± 2.45
P. aeroginosa	18.81 ± 1.24
ATCC 27853(*P.aeruginosa*)	17.85 ± 1.28
