# Supplementary material for: Stingless bee honey: Nutritional, physicochemical, phytochemical and antibacterial validation properties against wound bacterial isolates
Source: PLoS One. 2024 May 14;19(5):e0301201. doi: 10.1371/journal.pone.0301201 (PMC11093306; doi:10.1371/journal.pone.0301201)
Supplement: S6 Table — (PDF) [file pone.0301201.s012.pdf]

S6 Table. Minimum bacteriostatic and bactericidal concentration. Table 6.

	Staphylococcus aureus		*Escherichia coli*		*Pseudomonas aeruginosa*		*Klebsiella pneumoniae*	
	MIC µg/ml	MBC µg/ml	MIC µg/ml	MBC µg/ml	MIC µg/ml	MBC µg/ml	MIC µg/ml	MBC µg/ml
**MuM**	62.5	250	250	125	62.5	125	125	125
**MaM**	125	250	250	250	125	125	62.5	62.5
**KiM**	125	125	125	125	62.5	62.5	62.5	62.5
**KoM**	62.5	125	125	250	62.5	125	62.5	125

KEY: **MIC** – Minimum inhibition concentration, **MBC** – Minimum Bactericidal concentration. **MaM** – Maoi Meliponin, **MuM** – Mukutani Meliponin, **KiM** – Kibigor Meliponin, **KoM**- Koriema Meliponin).
