# Supplementary material for: Stingless bee honey: Nutritional, physicochemical, phytochemical and antibacterial validation properties against wound bacterial isolates
Source: PLoS One. 2024 May 14;19(5):e0301201. doi: 10.1371/journal.pone.0301201 (PMC11093306; doi:10.1371/journal.pone.0301201)
Supplement: Attachment — Submitted filename: 2022 PONE-D-22-28752_REV 1 22 Nov.pdf [file pone.0301201.s013.pdf]

PLOS ONE

Stingless bee honey: Nutritional, physicochemical, phytochemical and antibacterial validation properties against wound bacterial isolates

--Manuscript Draft--

**Manuscript Number:**	PONE-D-22-28752
**Article Type:**	Research Article
**Full Title:**	Stingless bee honey: Nutritional, physicochemical, phytochemical and antibacterial validation properties against wound bacterial isolates
**Short Title:**	Nutritional, physicochemical, phytochemical and antibacterial validation properties of stingless bee honey
**Corresponding Author:**	miriam Wanjiru mwangi, Masters degree<br>Egerton University - Njoro Campus: Egerton University<br>egerton, riftvalley KENYA
**Keywords:**	antibacterial; Stingless bee honey; antimicrobial resistance; PHYTOCHEMICALS
**Abstract:**	The discovery of new antibacterial compounds is paramount due to the raising challenge of bacterial resistance to the already existing arsenal of antibiotics. The nutritional, physicochemical, phytochemical and antibacterial properties of stingless bee honey collected from Marigat, Baringo County were analyzed. This was followed by analysis of its antibacterial activity against various isolated and characterized wound bacterial isolates. The inhibition obtained on each of the bacterial isolates against different concentrations of honey impregnated discs was compared to conventional antibiotic discs. Various components in honey contribute towards its antibacterial effectiveness which compares well with conventional antibiotics since most bacterial isolates indicate higher susceptibility. Data was analyzed using IBM SPSS statistics 23 and the variables obtained were significantly different ($P<0.005$) using Two Way ANOVA through graph pad prism version 8.0.2 statistical software.
**Order of Authors:**	miriam Wanjiru mwangi, Masters degree
	ERIC OMORI OMWEGA, PhD
	TABITHA W. WANJAU, PhD
**Additional Information:**	
**Question**	**Response**
**Financial Disclosure**<br><br>Enter a financial disclosure statement that describes the sources of funding for the work included in this submission. Review the submission guidelines for detailed requirements. View published research articles from *PLOS ONE* for specific examples.<br><br>This statement is required for submission and **will appear in the published article** if the submission is accepted. Please make sure it is accurate.	The authors received no specific funding for this work

Powered by Editorial Manager® and ProduXion Manager® from Aries Systems Corporation

**Unfunded studies**<br>Enter: *The author(s) received no specific funding for this work.*<br><br>**Funded studies**<br>Enter a statement with the following details:<br>• Initials of the authors who received each award<br>• Grant numbers awarded to each author<br>• The full name of each funder<br>• URL of each funder website<br>• Did the sponsors or funders play any role in the study design, data collection and analysis, decision to publish, or preparation of the manuscript?<br>• **NO** - Include this sentence at the end of your statement: *The funders had no role in study design, data collection and analysis, decision to publish, or preparation of the manuscript.*<br>• **YES** - Specify the role(s) played.<br><br>* typeset	
**Competing Interests**<br><br>Use the instructions below to enter a competing interest statement for this submission. On behalf of all authors, disclose any competing interests that could be perceived to bias this work—acknowledging all financial support and any other relevant financial or non-financial competing interests.<br><br>This statement is required for submission and **will appear in the published article** if the submission is accepted. Please make sure it is accurate and that any funding sources listed in your Funding Information later in the submission form are also declared in your Financial Disclosure statement.<br><br>View published research articles from *PLOS ONE* for specific examples.	The authors have declared that no competing interests exist

**NO authors have competing interests**<br><br>Enter: *The authors have declared that no competing interests exist.*<br><br>**Authors with competing interests**<br><br>Enter competing interest details beginning with this statement:<br><br>*I have read the journal's policy and the authors of this manuscript have the following competing interests: [insert competing interests here]*<br><br>* typeset	
**Ethics Statement**<br><br>Enter an ethics statement for this submission. This statement is required if the study involved:<br><br>• Human participants<br>• Human specimens or tissue<br>• Vertebrate animals or cephalopods<br>• Vertebrate embryos or tissues<br>• Field research<br><br>Write "N/A" if the submission does not require an ethics statement.<br><br>General guidance is provided below. Consult the submission guidelines for detailed instructions. **Make sure that all information entered here is included in the Methods section of the manuscript.**	Written consent was given IREC (UEAB / 7/12/2016 and NACOSTI (NACOSTI / P/22/18002)

Powered by Editorial Manager® and ProduXion Manager® from Aries Systems Corporation

**Format for specific study types**<br><br>**Human Subject Research (involving human participants and/or tissue)**<br>• Give the name of the institutional review board or ethics committee that approved the study<br>• Include the approval number and/or a statement indicating approval of this research<br>• Indicate the form of consent obtained (written/oral) or the reason that consent was not obtained (e.g. the data were analyzed anonymously)<br><br>**Animal Research (involving vertebrate animals, embryos or tissues)**<br>• Provide the name of the Institutional Animal Care and Use Committee (IACUC) or other relevant ethics board that reviewed the study protocol, and indicate whether they approved this research or granted a formal waiver of ethical approval<br>• Include an approval number if one was obtained<br>• If the study involved *non-human primates*, add *additional details* about animal welfare and steps taken to ameliorate suffering<br>• If anesthesia, euthanasia, or any kind of animal sacrifice is part of the study, include briefly which substances and/or methods were applied<br><br>**Field Research**<br><br>Include the following details if this study involves the collection of plant, animal, or other materials from a natural setting:<br>• Field permit number<br>• Name of the institution or relevant body that granted permission	
**Data Availability**<br><br>Authors are required to make all data underlying the findings described fully available, without restriction, and from the time of publication. PLOS allows rare exceptions to address legal and ethical concerns. See the PLOS Data Policy and FAQ for detailed information.	Yes - all data are fully available without restriction

Powered by Editorial Manager® and ProduXion Manager® from Aries Systems Corporation

A Data Availability Statement describing where the data can be found is required at submission. Your answers to this question constitute the Data Availability Statement and **will be published in the article**, if accepted.<br><br>**Important:** Stating 'data available on request from the author' is not sufficient. If your data are only available upon request, select 'No' for the first question and explain your exceptional situation in the text box.<br><br>Do the authors confirm that all data underlying the findings described in their manuscript are fully available without restriction?	
**Describe where the data may be found in full sentences. If you are copying our sample text, replace any instances of XXX with the appropriate details.**<br><br>• If the data are **held or will be held in a public repository**, include URLs, accession numbers or DOIs. If this information will only be available after acceptance, indicate this by ticking the box below. For example: *All XXX files are available from the XXX database (accession number(s) XXX, XXX.*).<br>• If the data are all contained **within the manuscript and/or Supporting Information files**, enter the following: *All relevant data are within the manuscript and its Supporting Information files.*<br>• If neither of these applies but you are able to provide **details of access elsewhere**, with or without limitations, please do so. For example:<br><br>*Data cannot be shared publicly because of [XXX]. Data are available from the XXX Institutional Data Access / Ethics Committee (contact via XXX) for researchers who meet the criteria for access to confidential data.*<br><br>*The data underlying the results presented in the study are available from (include the name of the third party*	Data are all contained within the manuscript and / or supporting information files

and contact information or URL).<br>• This text is appropriate if the data are owned by a third party and authors do not have permission to share the data.<br><br>* typeset	
Additional data availability information:	Tick here if your circumstances are not covered by the questions above and you need the journal’s help to make your data available.

Manuscript

Click here to access/download;Manuscript;MANUSCRIPT I-(JULY 22).docx

Stingless bee honey: Nutritional, physicochemical, phytochemical and antibacterial validation properties against wound bacterial isolates

Mwangi Miriam Wanjiru[1*], Eric Omori Omwenga[2ж] and Tabitha W. Wanjau[1 ж]

[1] Department of Applied Sciences, School of Health Sciences, Kisii University, P.O. Box 408-40200, Kisii, Kenya.
[2] Department of Medical Microbiology & Parasitology, School of Health Sciences, Kisii University, P.O. Box 408-40200, Kisii, Kenya.

ж These authors contributed equally to this work

* **Corresponding Author**: Email; mwangimirriam6@gmail.com. Phone number: - +254 0722398956

Data Availability: All relevant data are within the manuscript

Funding: The authors received no specific fund for the research of this work

Competing Interests: The authors have declared that no competing interests exist

ABSTRACT

With the rise of AMR the management of wound infections are becoming a big challenge. This has been attributed to the fact that most wound bacterial isolates have been found to possess various virulence factors like enzymes, toxins & biofilms production. Therefore, need for discovery of new lead compounds is paramount as such factors make these microbes to be resistant to already existing arsenal of antibiotics or even the immune system. This study aimed at documenting the nutritional, physicochemical, phytochemical and antibacterial properties of stingless bee honey. Isolation and characterization of bacterial isolates from 34 samples obtained from wounds of outpatients and surgical wards of Nakuru County Referral Hospital, Kenya was done. Various bacterial isolates (43) were isolated - *Staphylococcus aureus* (34.8%) being predominant, followed by *Pseudomonas aeruginosa* (27.9%), *Klebsiella pneumoniae* (23.3%) and *Escherichia coli* (14.0%). A total of 36 out of the total isolates were genotypically characterized using molecular techniques detecting the prevalence of the following virulence genes; 16SrRNA (756bp), hla (229bp), cnf1 (426bp), cnf2 (543bp), hlyA (1011bp), rmpA (461bp), lasL (600bp), gyrB (411bp), khe (77bp) and magA (128bp). An assessment of the *in vitro* antibacterial activity of 26 stingless bee (*Meliponines spp.*) honey samples collected from their resin egg-shaped pots in Marigat sub County, Baringo County, Kenya was done. Antibacterial properties of the stingless bee honey was done with varying susceptibility patterns being observed at different concentrations of honey impregnated discs ($10x10^4$, $20x10^4$, $50x10^4$ and $75x10^4$ µg/ml) giving mean inhibition diameters of 18.23 ± 0.4mm (*Staphylococcus aureus)*, 17.49 ± 0.3mm (*Pseudomonas aeruginosa*), 16.05 ± 0.6mm (*Klebsiella pneumoniae)* and 10.19 ± 0.5mm *(Escherichia coli)* with a mean range of 14.54 ± 2.0mm to 17.58 ± 3mm. Higher susceptibility to honey was recorded across all the bacterial isolates compared to conventional antibiotics while the mean MIC and MBC of the honey were recorded at 62.5µg/ml and 250µg/ml respectively. Control bacterial isolates *Staphylococcus aureus* ATCC 25923, *Escherichia coli* ATCC 25922, *Klebsiella pneumoniae* ATCC 27736 and *Pseudomonas aeruginosa* ATCC 27858 were used in the analysis. The stingless bee honey was found to be rich in various nutritive components like sugar (89.85 ± 5.07 g/100g) and moisture (81.75 ± 10.35 mg/g) with a significant difference of $P < 0.05$ as the main antibacterial components. Additionally, the stingless honey did possess water soluble vitamins, proteins and minerals of which potassium was the most dominant one. In regard to phytochemicals, on our preliminary analysis phenolic, flavonoid and carotenoid compounds were

found to be present with phenolic compounds being the most dominant one. Stingless bee honey from Marigat, has antimicrobial properties which could be attributed to the rich phytochemicals it possesses and its physicochemical properties in addition to its high nutritive value.

Key words: Antibacterial, Stingless bee honey, Antimicrobial resistance, Phytochemicals

Author Summary

The surge in antibiotic resistance in recent decades is primarily attributed to excessive and inappropriate use of antibiotics in both human and animal welfare, over-the-counter availability of antibiotics, poor sanitation and hygiene and environmental recycling of non-metabolized antibiotics though human and animal consumption. It is with this understanding that we decided to explore the antibacterial therapeutic value existing in stingless bee honey collected from Marigat, Baringo County, Kenya. We first analyzed the phytochemical, physicochemical and nutritional components that confer the antibacterial effectiveness in honey. The honey in different dilutions was then subjected against bacterial isolates from cutaneous wounds and burns. We were also able to make a comparison between honey and conventional antibiotics in their antibacterial effectiveness.

1.0 INTRODUCTION

Wounds represents disorder to an organ or a tissue involving devitalized and missing cellular structures and tissue layers [1] They may spread to other anatomical structures and the presence of proliferating microbes cause cellular hence inflammatory response enough to hinder the healing process [2]. In the United States chronic wounds affects between 1.5% and 3% of the general population, 15% to 20.3% in China, with about 14.8% overall incidences being reported in Sub-Saharan Africa, whereas in Kenya most injuries are implicated during drought seasons, intertribal crushes, cattle rustling as well as attacks by wild animals with the incidence rate of surgical site and post-caesarean wound infections of 7.0% and 19% respectively [3]. Wounds persistence in Kenya is enhanced by high treatment cost and high poverty levels as well as use of other medications like corticosteroids and chemotherapeutic [4].

Complicated wound and other infection management is caused by increase in emergence of drug and multidrug resistant microorganisms due to the over use and misuse of antibiotics which normally cause toxic side effects to humans. [5] Furthermore, less new generation of antibiotics have been produced in the last decade with postulations that by 2050 we may be in a world with no active antibiotics [6] hence, need to explore nature for new remedial measures to curb the upsurge of the super resistant bugs. Despite all this, the local populations have been applying various traditional

methods like medicinal plant use, honey use etc. to manage wounds for decades [7]. Since the ancient times humans have used honey in management of wound infections [9]. Honey has also been found to possess antibacterial properties that do vary from one region to another based on previous studies [10]. These differences in terms of their antimicrobial activities have been attributed to differences in their chemical composition and physicochemical properties that have been found to differ according to the nectar origins, bee species and other intrinsic factors like osmotic effect, acidity, etc. [11]. Similar to the honey bees, the stingless bees collect nectar for honey production but store it in honey pots made of cerumen (contains the mandibular secretions) instead of hexagonal honeycombs attributed to the good quality of honey [12]. Based on this therefore, the current study aimed at deducing the antibacterial efficacy of honey from stingless bees that is commonly used by inhabitants of Baringo County, Kenya. The study further sort to validate the nutritional, physicochemical and phytochemical of the stingless bee honey.

2.0 MATERIALS AND METHODS

2.1 Study area

The study sampled the stingless bee honey from the semi-arid rangeland in Marigat sub County, Baringo County, Kenya (Figure 1) that is a home for most acacia, low trees and shrubs (latitude $00^o26'$ to $00^o32'$N, longitude $36^o00'$ to 36^o09E and altitude 900 to 1,200 M above the sea level, agro-climatic zones IV &V, annual temp. & rainfall of 24.6^o C & 671mm), according to Koppen and Geiger [13].

2.2 Sample Size Determination and Sampling Techniques

2.2.1 Wound Sampling Techniques

Wound swabs were obtained from out patients as well as hospitalized surgical wards patients at County Referral Hospital- Nakuru with an informed patients' consent (supplementary data S3) and established protocols as used before [14]. A swab was taken from every patient within inclusion criteria prior to being transported to the laboratory in Stuart transport medium. The sample size determination method for wound swabs employed for the target population was used as applied by [15] that ended up producing a sample size of 38 swabs as indicated below;

$$S = \frac{Z^2 X(P) X(1-P)}{C^2}$$

Where; Z – Z value (e.g. 1.96 for 95% confidence level)

P - Percentage picking a choice, expressed as a decimal

C - Confidence Interval

Hence; $\frac{(1.96)^2 x(0.5) x(1-0.5)}{(0.025)^2} = 38$

2.2.2 Honey Sampling Techniques

Stingless bees' hives were placed in a dark room and the honey from their honey pots removed by sacking with a syringe [16]. The sample size determination method for honey samples employed for the target population was as used by [17] and a sample size of 26 honey samples was obtained and used for this study as indicated below;

$$N = \frac{Z^2 XPXQ}{D^2}, \quad N = \frac{(1.96)^2 X \left(\frac{1500}{19895}\right) X1 - P)}{(0.05)^2} = \frac{103}{4} = 26$$

2.3 Analysis of the physicochemical properties of the honey samples

Analysis was done in accordance with [18] as shown below.

2.3.1 Determination of sugar content

Spectrophotometric method were used as previously applied [19]. 2.0g of the honey sample was mixed with 10ml dimethyl sulfoxide (DMSO) (25% v/v) and incubated in a water bath at 100°c for 20minutes. 0.5ml of the mixture was diluted with 9.5ml distilled water followed by addition of 0.5ml of phenol (5%) and 2ml of 75% sulphuric acid (H_2So_4), finally absorbance was read at 492nm against a standard glucose solution.

2.3.2 Determination of moisture in honey samples

The determination of moisture was done using a method described by [18]. Briefly, 2.0g of honey samples were weighed out and dried to a constant weight in a hot air oven at 70°c and then cooled

down and weighed again (oven-dry moisture content). The difference in the dry weight gave the moisture content.

2.3.3 Determination of pH in honey samples

To determine the pH of honey, a digital pH meter calibrated at pH 4 and 10 was applied as used before [20]. Briefly, working solution of honey was made by mixing 10g of honey in 75ml of distilled water and using the calibrated pH electrode the pH reading on the meter was recorded.

2.3.4 Determination of titratable acidity in honey samples

The titratable acidity of the honey was determined using well established protocols as used before [21]. 10g of honey was dissolved in 75ml of distilled water, the calibrated reference electrode was immersed and titrated to pH 8.5 with 0.05M NaOH at a rate of 5ml/minute and recorded. ~~10ml of 0.05M NaOH was added into the solution and back titrated to pH 8.3 using 0.05M HCL from a second burette and recorded~~. A blank determination procedure of 75ml of distilled water was also run to pH 8.5, the free acidity is the acidity titrable with sodium hydroxide up to the equivalent point.

2.3.5 Determination of Hydroxy methyl furfural (HMF)

The spectrophotometric method involves measurement of UV absorbance of honey solutions as used before [20]. 5g of honey was weighed and dissolved in 25ml distilled water into a 50ml volumetric flask. 0. l5 ml of Carrez solution I and 0.5 ml Carrez solution II were added and made up to 50ml with distilled water, then filtered and after rejecting the first 10ml of the filtrate the aliquots of 5ml were put in two test tubes. 5ml of distilled water and 5ml of fresh 0.2% sodium metabisulphite were put into sample solution and reference solution tubes respectively. The absorbance was read at 284nm and 336nm using a UV-Visible mini ± 1240 Shimadzu spectrophotometer**.**

2.3.6 Determination of hydrogen peroxide in honey

This was done using a method used by [22]. Briefly, 30% (w/v) concentration of honey at pH8 by adjusting the pH metre was made followed by incubation in a water bath at 37°c for 30minutes. Hydrogen peroxide specific test strips were immersed into the solution for one second, the excess

solution was shaken off the strip and the colour developed read against a colour code to obtain the concentration of hydrogen peroxide in the honey samples.

2.4 Nutritive Analysis of Honey

2.4.1 Determination of sugar content

The sugar content determination was carried out using standard procedures as used by [16]. Briefly, 2.0g of the honey sample was mixed with 10ml dimethyl sulfoxide (DMSO) (25% v/v) and incubated in a water bath at 100°c for 20minutes. 0.5ml of the mixture was diluted with 9.5ml distilled water, followed by 0.5ml of phenol (5%) and 2ml of 75% sulphuric acid (H_2So_4) and the absorbance read at 492nm against a standard glucose solution.

2.4.2 Determination of Honey Protein Concentration

It was done according to Kjeldahl method as used by [23]. 0.5grams of honey was transferred to the labelled kjeldahl tubes, with 2.5gram of the catalytic mixture and 7ml sulphuric acid. The mixture was placed in a block digester with gradual temperature increase (50°c – 400°c) for 5hours followed by heating the water to boil for 10minutes and finally dissolved in 10ml distilled water. To a 125ml Erlenmeyer flask, 15ml of boric acid (5%) was put followed by 5drops of the mixed indicator (Methyl red and Bromocresol green) which is red for acidic and green for basic, 20ml of sodium hydroxide (50%) was then added to the digestion tubes until the samples were neutralized to a dark blue colour.50 ml of standard hydrochloric acid (0.01M) was put in a burette and titrated directly into the Erlenmeyer flask, pink colour indicated end-point and the volume of HCL used determined the protein concentration.

2.4.3 Determination of Vitamin C Content

It was carried out using standard procedures as used before [24]. 100mg of honey sample was put into a flask followed by addition of 10ml of metaphosphoric acid 1% and filtered through Whatman filter paper No. 4. Then 1ml of the filtrate was mixed with 9ml 2, 6- dichlorophenolindophenol (DCPIP) 0.005% and the absorbance analyzed spectrophotomically within 30minutes at 515nm.

2.4.4 *Determination of Water - Soluble Vitamins in Honey (B_2, B_3, B_5 and B_9)*

The determination among the samples was carried out using the standard procedures as used before [25]. Briefly, 10gram of the honey sample was dissolved through stirring in 10ml distilled water followed by addition of 1ml of sodium hydroxide (2M) , 12.5ml of phosphate buffer 1M (pH5.5) and then topped up to the 50ml mark in a volumetric flask with distilled water. The sample solutions were injected through the filter in a spectrophotometer at the selected wavelengths i.e. VitB2- 210nm, VitB3 – 254nm, VitB5 – 210nm and VitB9 – 210nm and the triplicate readings recorded.

2.4.5 Determination of Calcium, Magnesium, Iron and Zinc in Honey

An Atomic Absorption Spectrometer (AAS) as used as before [26]. 10gram of honey was heated to 500°c to obtain a constant dry weight with an infrared lamp, in order to prevent foaming. The ash samples formed were dissolved in 10ml perchloric acid (60%) and 10ml nitric acid (65%), filtered and individual minerals determined using an air – acetylene flame and a hollow cathode lamp using known standards and wavelengths (Copper- 324nm, Iron – 248nm, magnesium- 285nm and Zinc – 213nm) for each individual mineral.

2.4.6 Determination of Sodium and Potassium in Honey Samples

The standard procedures as used before [27] were applied where 5 gram of honey was put into a 15ml polypropylene flask then 0.4ml of caesium chloride solution (5% m/v) and 10ul of hydrochloric acid were added followed by N – Propanol up to the 10ml final volume. Internal standards were prepared by diluting ethanol and yttrium (1: 10 m/v) and absorbance measured out in the flame emission mode under different wavelengths (766nm and 589nm for potassium and sodium) specific for the minerals.

2.4.7 Determination of Phosphorous in Honey Samples

This was done in accordance to the described method as done before [27]. 10gram of honey was put into a porcelain crucible and dissolved in 5ml nitric acid (1N) in tubes, heated in a water bath for 3minutes followed by dissolving the mixture in distilled water up to 100ml and filtered. 5ml of the solution was put into 100ml volumetric flask followed by addition of 10ml of phosphate standard (0.1mg/ml), 10ml of nitric acid (6N), 10ml of ammonium molybdate (0.2%), and 10ml of ammonium molybdate (5%) and finally diluted to the 100ml mark with distilled water and allowed to stand for 15minutes at room temperature to allow complete color development then absorbance was measured at 400nm wavelength against a reagent blank for auto-zero and then recorded.

2.5 Determination of Phytochemical and Antioxidant Properties of Honey

2.5.1 Total Content of Phenolic Compounds (TCPC)

This applied a method by [28], 5 grams of each honey sample was diluted to 50ml with distilled water, mixed and filtered, followed by addition of 2.5ml of 0.2N folin-ciocalteu and incubated at room temperature for 5minutes. 2ml of 75g/l sodium carbonate ($NaCo_3$) was then added and incubated again at room temperature for two hours in the dark and absorbance read at 760nm against a methanol blank in spectrophotometer. Gallic acid was used as a reference standard and results expressed as mg gallic acid equivalent (mgGAE).

Flavonoids was determined by use of a method that was used before [29], 0.5 grams of honey was mixed with 5ml of methanol (50%) and filtered, 5ml of the honey solution was mixed with 5ml of 2% aluminium chloride ($AlCl_3$) and incubated for 30minutes at room temperature and absorbance read at 420nm using UV Visible spectrometer and expressed in milligram of rutin equivalent (RE) in grams of honey

The total content of carotenoids was determined by use of well-established protocol as used by [23]. 1gram of the honey sample was mixed with 10ml of n-hexane acetate mixture and homogenized thoroughly for 10minutes at room temperature then allowed to stand in the dark for 30minutes, filtered and absorbance of the filtrate measured at 450nm using a spectrophotometer. The total carotenoids content was expressed as mg of β – carotene equivalents (mg β- carotene /kg of honey).

2.5.2 Determination of Vitamin C (ascorbic acid) Content

The determination was by use of well-established protocol as used by [23]. To 100gram of the honey sample, 10ml 1% metaphosphoric acid was added at room temperature, filtered then1ml of the filtrate was mixed with 9ml 2,6, dichlorophenolindophenol (DCPIP) 0.005% and the absorbance measured within 30minutes at 515nm.

2.6 Quality Assurance

Quality Control (QC) measures such as use of known standards, laboratory synthesized honey used as a negative control, the International Control Bacterial strains from American Type Culture Collection (ATCC), *Escherichia coli* (ATCC 25922), *Klebsiella pneumoniae* (ATCC 27736),

Pseudomonas aeruginosa (ATCC 27853) and *Staphylococcus aureus* (ATCC 25923) were included in the study according to the Clinical and Laboratory Standards Institute (CLSI).

2.7 Isolation and Characterization of wound microorganisms

2.7.1 Phenotypic detection

The suspected micro-organisms in the wound swabs were isolated through streaking on sterile agar plates for the identification and of pure microbial colonies. The evaluation of the morphological features together with Gram stain and biochemical tests (Coagulase, Methyl red, TSI and Oxidase tests) enhanced their further identification [30]. MacConkey and CLED agar were used in obtaining pure bacterial colonies which were then differentiated as Gram positive or negative upon staining.

2.7.2 Molecular detection through characterization of specific virulence genes

i. DNA Extraction and Amplification

This was done by use of the Fungi / Bacteria DNA premix kit from Zymo, USA, according to [31] protocol. The unbiased mechanical lysis of the microbes was achieved by bead beating with the ultrahigh density bashing beads then the debris were filtered from lysate using Zymo-spin. Washing followed to elute the solution from the Zymo-spin followed by complete PCR inhibitor removal by filtering the eluate with a Zymo-spin giving purified DNA ideal for microbial community profiling.

ii. PCR- based screening of virulence genes

This is a technique that makes multiple copies of a short sequence of target DNA by amplifying it in order to make enough material for detection and identification with a high degree of confidence by a fluorescence signal. DNA fragments of the pre-detected size were amplified by PCR primers with an amplification specific to the gene in question. Electrophoresis technique was used as before [32] to identify, quantify and purify nucleic acid fragments through loading the samples into wells on agarose and subjecting it to an electric field. The negatively charged nucleic acids move to the positive electrode, shorter fragments travel rapidly while longest fragments remain close to the origin of the gel resulting in separation based on size. Ethidium bromide DNA stain was used to enhance detection sensitivity while a 100bp DNA ladder was used for quantitative analysis of linear double-stranded DNA in agarose gels by indicating the base pair (bp) length of nucleic acids. The virulent genes for detection specific to the bacterial isolates included; *Staphylococcus aureus* (Hla and 16SrRNA),

Escherichia coli, (CNF1, CNF2 and HlyA), *Klebsiella pneumoniae (*Khe, RmpA and MagA) and *Pseudomonas aeruginosa* (LasL and GyrB).

2.8 Antimicrobial activity determination of honey compared to conventional antibiotics

2.8.1 Preparation of honey discs

50µl of various dilutions of stingless bees honey samples ($10x10^4$, $20x10^4$, $50x10^4$ and $75x10^4$ µg/disc) were impregnated on Whatman No. 1 filter paper discs (6mm) and allowed to dry under sterile conditions.

2.8.2 Disc diffusion method

The disc diffusion (Kirby-Bauer) technique as used before [33] was employed using Muller- Hinton agar (Hi-Media, India), which was prepared according to the manufacturer's instructions. An inoculum prepared by emulsifying two colonies of the isolates in 10ml distilled water and adjusting the turbidity to $1.5x10^8$ CFU/ml. (According to the McFarland Standards) was inoculated on Muller-Hinton agar through pour plating as done before [34 & 35] after 15minutes, the discs with test honey, reference and artificial samples were placed on the plates and incubated at 4°C for 2hours to allow pre-diffusion of honey into the media, then at 37°C for 24 hours and the diameter of each zone of inhibition measured. Cartridges containing commercially available antibiotics including Levofloxacin (5µg), Ampicillin (10µg), Tazobactum (110µg), Meropenem (10µg), Gentamycin (10µg) and Chloramphenicol (30µg) were also set against the bacterial isolates. Sterile Whatman No. 1 plain filter paper discs were also set on the plates as negative control.

2.8.3 Evaluation of the bacteriostatic and bactericidal activity of honey

Bacteriostatic and bactericidal properties of the honey samples were determined according with the micro dilution method as used by [36 & 37]. 5ml of nutrient agar was put in 8tubes, 5mg (5000µg) of honey was added to tube 1 and mixed well by vortexing to make a concentration of 1000µg/ml. This was serially twofold diluted using nutrient agar up to tube number 6 to obtain various ranges of concentration between 250µg/ml and 31.25µg/ml. Tube 7 was labelled GC and no honey was added. A volume of 100µg/ml of the bacterial inoculum was added into all tubes except tube 9 (HC) followed by incubation at 37°C for 24hours followed by physical examination for turbidity. The tubes containing the least concentration showing no visible growth was considered as MIC and the previous

lower dilution tube were inoculated onto sterile nutrient agar plates by the streak plate method and incubated aerobically at 37^0C for 24 hours. The least concentration that did not showed any growth was considered as the MBC.

2.9 Data analysis

The data in this study was first processed using IBM SPSS (Statistical Package for Social Sciences) statistics 23 and Two – way ANOVA through the Graph Pad Prism (Version 8.0.2-263) statistical software was used to compare mean values among the various experiments and to analyze their significant difference (P values < 0.005). Variables were considered significant by * and highly significant when P<0.01(**), P < 0.001 (***), P< 0.0001 (****).

2.10 Approvals

To ensure the required quality assurance, this study was approved by IREC (Ethics clearance for research proposal) and NACOSTI (National commission for science, technology and innovation) as attached in supplementary data S1 and S2 respectively.

3.0 RESULTS

3.1 Characterization and identification of bacteria

3.1.1 Culturing characteristics

Various colonial morphologies were observed as; Large, round, convex, sharp border colonies with uniform golden – yellow colour on CLED agar and those surrounded by clear zones of beta haemolysis on blood agar suggestive of *Staphylococcus aureus*, opaque, yellow lactose fermenting colonies with a slightly deeper yellow center on CLED agar suggestive of *Escherichia coli,* yellow to whitish – blue, extremely mucoid colonies on CLED agar as well as mucoid lactose fermenting colonies with a convex elevation on MacConkey suggestive of *Klebsiella pneumoniae* and colorless to pink, flat, irregular colonies with alligator skin like surface and typical grape –like odour observed suggestive of *Pseudomonas aerugin*osa as indicated in supplementary data S4 attached. A total of 28 burn and wound swabs (82.35%) yielded notable colonies of bacterial growth indicative of wound infection while 6 samples (17.6%) did not yield any colonies indicative of growth. According to the analysis, 15 (44.1%), 11(32.4%) and 2 (5.9%) wound swabs yielded 1, 2 and 3 isolates respectively hence giving rise to a total of 43 pure isolates as shown in Table 1 attached.

3.1.2 Gram stain

Various Gram reactions were realized from the isolated bacterial isolates as; Gram positive (purple) cocci shaped bacteria in clusters ("grape – like") suggestive of *Staphylococcus aureus,* Gram – negative pink-red rods (singly and in pairs) suggestive of *Escherichia coli,* Pink-red rod- shaped bacilli (singly, pairs, short chains and capsulated) suggestive of *Klebsiella pneumoniae* and Gram – negative rod-shaped bacilli that appeared slimmer and pale staining than members of *Enterobacteriaceae* as shown in supplementary data S5 and table 2 attached.

3.1.3 Biochemical reactions

From the samples isolated in this study, 35% tested positive for coagulase test a possible positive indication of the presence of *Staphylococcus aureus*. When the samples were subjected to Triple sugar iron agar (TSI) 21% of the samples tested positive, an acidic slant (yellow) and an acidic butt (yellow) were observed indicating that the isolate was glucose, lactose and /or sucrose fermenter. Bubbles in the tube indicated production of carbon dioxide as a product of fermentation as a clear indication that the isolate could be *Escherichia coli* as shown on supplementary data 5 and table 3 attached. Methyl red was also used to confirm presence of pathogens that do not ferment glucose. From our findings 46% of the isolates formed a red ring indicating a methyl red positive result, a finding that demonstrates the presence of *Escherichia coli*. Those that proved to be negative for this test (55%) was indicative of *Klebsiella pneumoniae* and finally, Oxidase test was done to differentiate *Pseudomonas aeruginosa* from other bacteria like *Escherichia coli* and it was deduced that 28% of the samples tested positive for the presence of *Pseudomonas aeruginosa* as indicated in supplementary data 5 and table 3 attached.

3.1.4 Molecular identification of bacteria through amplification of virulence genes

In this study, molecular techniques (PCR assays and gel electrophoresis) were used to reveal the prevalence of 10 virulence genes including 16srRNA (756bp), hla (229bp), cnf1 (426bp), cnf2 (543bp), hlyA (1011bp), rmpA (461bp), LasL (600bp), gyrB (411bp), khe (77bp) and magA (128bp) using appropriate primer sequences. According to figure 2 attached, a total of 36 out of 43 isolates (84%) indicated varying amplicons; 42% of *Staphylococcus aureus* (16srRNA – 756bp and hla – 209bp - lane 1 and 2), 7% of *Escherichia coli* (cnf1- 498bp, hly – 1177bp),17% of *Klebsiella pneumoniae* (magA - 121bp, rmpA 106bp) and *Pseudomonas aeruginosa* (gyrB 222bp and lasL

600bp genes - lanes 12 and 13). Cnf2 and khe indicated no amplicons (lanes 4, 5, 8, 9, 10). Control organisms were also analyzed as a confirmatory for the presence of the virulence genes.

3.2 Physicochemical and Nutritive properties of honey

The findings of physicochemical and nutritive properties were compared to the standards [38].

3.2.1 Hydromethylfurfural (HMF)

A high HMF mean concentration (22.77 ± 13.6mg/kg) was detected and was within the required range according to the International Regulations of quality [48] of 5mg/kg to 80mg/kg for honeys coming from tropical temperatures. There were significant differences between the samples (P<0.05) as shown in figure 3 attached.

3.2.2 Sugar concentration

Mean sugar content of the stingless bee honey samples was 89.85± 6.0 g/100g which was within the recommended values of 60g/100g to 800g/100g [39]. Samples from the arid semi-arid and medium altitude regions indicated varying mean sugar concentrations of 94.0 ± 1.26 g/100g and 86.0 ± 5.02 g/100g. The values were significantly different across the regions (P<0.05) as indicated in figure 3 attached.

3.2.3 Moisture

The stingless bee honey samples indicated a moisture mean content of 81.75 ± 10.4mg/g which is in accordance as recommended by the [39] with a significant difference among of the honey samples across the different regions (P<0.05) as illustrated in figure 3 attached.

3.2.4 pH and Free acidity

The stingless honey samples had a mean pH value of 3.86 ± 0.11 and within the recommended limit (3.2 to 4.5) according to [39]. The mean free acidity was 0.063 ± 0.0, which was a bit low but within the recommended levels (<50meq/kg) according to [38]. There were no significant differences in free acidity and pH between them (P>0.05) as indicated in figure 3 attached.

3.2.5 Hydrogen peroxide

Hydrogen peroxide mean concentration of 1 ± 0.41mM was recorded with highest mean concentration of 1.25 ± 0.35mM recorded from Mukutani region, and were within the recommended range of 0.5 and 2.5mM according to [38]. There was no significant difference samples from different regions as indicated in figure 3 attached.

3.2.6 Water Soluble Vitamins

Water soluble vitamins found in stingless bee honey include Vitamin B1, B2, B3, B5 and B9 (0.21 ± 0.03 to 0.64 ± 0.02mg/l, 0.72 ± 0.03 to 2.39 ± 0.07 mg/l, 0.65 ± 0.5 to 4.19 ± 0.02 mg/l, 0.01 ± 0.0 to 1.22 ± 0.03mg/l and 0.11 ± 0.00 to 1.64 ± 0.10 mg/l) which were within the Limit of Detection approved by the international Union of Pure and Applied Chemistry (1.10 mg/kg to 1.75mg/kg). Vitamin B_2, B_3 and B_9 were significantly different ($P<0.05$) as in figure 4 attached.

3.2.7: Minerals

The stingless bee honey indicated high mean levels of calcium, magnesium, iron, sodium and potassium as 2.11 ± 0.20mg/l, 0.60 ± 0.47mg/l, 0.76 ± 1.84 mg/l, 1.24 ± 0.95mg/l and 17.94 ± 1.7 mg/l respectively which was lower than the recommended limit [44] of 3.23 to 236.8mg/l, 4.85 to 218.0mg/l, 2.18 to 563.72 mg/l, 16.66 – 1249.34mg/l and 0.41 – 224.0mg/l. There was significant difference ($P<0.05$) for Calcium, Magnesium and Zinc but non for Potassium, Iron, Sodium and Phosphorous as shown in figure 5 attached.

3.2.8: Crude proteins

The honey samples contained detectable amounts of proteins giving an average value of 1.33 ± 0.89g/100g which were slightly higher than the recommended range of 0.9 to 0.5g/100g according to the [39]. The protein content of the honey samples were significantly different ($p<0.05$) as shown in figure 5 attached.

3.3 Phytochemical properties of honey

The phytochemical components of stingless bee honey analyzed (Total flavonoid content, Total phenolic content, Vitamin C and Total carotenoid content) were expressed as mean range ± SD (mean average± SD) and the findings compared to the Standards of [38], as indicated in figure 6. The honey

samples indicated a higher mean Total Phenolic, Flavonoid and Carotenoid contents (92.18 ± 51.18mgGAE/100g, 23.7 ± 5.87mgRE/100g and 6.57 ± 0.21 mgβcarotene) as indicated in figure 6 attached.

3.4 Antibacterial activity of honey samples and selected antibiotics against wounds and burns bacterial isolates

3.4.1: Disc diffusion

The antibacterial activity of stingless bee honey sample was compared with those of commonly used antibiotics (Levofloxacin (5 µg), Ampicillin (10 µg), Tazobactam (110 µg), Meropenem (10 µg), Gentamicin (10 µg) and Chloramphenicol (30 µg)), by use of sterile discs impregnated with varying concentrations ($10x10^4$, $20x10^4$, $50x10^4$ and $75x10^4$ µg/disc) of the stingless bee honey samples. The antibacterial potency of the honey samples was indicated by production of clear zones in concentrations of $50x10^4$ µg/disc and $75x10^4$ µg/disc with minimal inhibition or none for the concentrations of $10x10^4$µg/disc and $20x10^4$µg/disc. *Staphylococcus aureus* was more susceptible followed by *Pseudomonas aeruginosa* then *Klebsiella pneumoniae* and finally, *Escherichia coli* shown in table 4 and figure 7 attached.

The study findings also did indicate that stingless bee honey had broad-spectrum antibacterial effect on the wound bacterial isolates. This was indicated by their susceptibility to the honey samples which compared well to the control bacterial isolates affirming its antibacterial effect against both Gram-positive and Gram-negative bacterial isolates. According to the findings of this study, the average inhibitions of all the stingless honey samples dilutions against the bacterial isolates compared well to the control bacterial isolates (ATCC) as tabulated in table 5 attached.

The bacterial isolates recorded varying susceptibility to the different antibiotics under study at; 9.50 ± 0.23mm to 25.0 ± 0.62mm, 6.0 ± 0.0 mm to 24.0 ± 0.56mm, 6.0 ± 0 .0 mm to 19.0 ± 0.50mm and 6.0 ± 0.0mm to 18.0 ± 0.46mm for *Staphylococcus aureus*, *Escherichia coli*, *Klebsiella pneumonia*e and *Pseudomonas aeruginosa* respectively. *Staphylococcus aureus* showed the highest susceptibility against most of the antibiotics but most isolates recorded considerable resistance except to Gentamicin (10µg) which indicated high inhibitory activity as indicated in figure 7.

3.5 Bacteriostatic and bactericidal activity of honey

The study evaluated the in vitro antibacterial activity (bacteriostatic and bactericidal effects) of stingless bee honey against four different bacterial cultures as wound isolates. The minimum inhibitory concentration for the honey samples was determined by micro dilution method indicating the inhibitory concentration at which stingless bee honey samples showed no visible growth of any test micro-organisms.

The MIC reflects the quantity needed for bacterial inhibition whose activity of the stingless honey samples against all the isolates was shown at 6.25 µg/ml while the MBC activity was at 250µg/ml. The findings did show the honey samples have an optimum bacteriostatic activity on the test isolates showing its antibacterial activity at 125µg/ml as shown in table 6 below with *Escherichia coli* proving to be the most resistant isolate since it indicated the highest MIC activity at concentration of 125µg/ml and was killed completely at higher concentrations of 250µg/ml. Therefore, the honey's antibacterial activity (bacteriostatic and bactericidal effect) similar to antibiotics was ascertained.

4.0 DISCUSSION

Microbial load in wounds cause delayed healing progress as they thrive well in hypoxic, non-healing wounds characterized by death and tissue necrosis [40]. This study identified 43 bacterial isolates from 34 wound swabs, *Staphylococcus aureus* was documented to be the most dominant isolate and was attributed to the fact that it forms the bulk of the normal skin flora followed by *Escherichia coli*, *Pseudomonas aeruginosa* and *Klebsiella pneumoniae* which was similar to other studies done by [41, 42, 43, 44 & 45]. These bacterial isolates are among the most notorious wound infections causing bacterial strains upon entry into wound tissues. They can complicate illness, anxiety, increase patient discomfort, result in a lengthy hospital stay and can also lead to death [46]. Majority of them are facultative anaerobes and this makes them thrive well in wound environment that has less levels of oxygen supply hence can induce an infection [47].

The isolates obtained from this study were found to be in possession of various virulence genes. For instance, all 15 S*taphylococcus aureus* isolates expressed the Hla gene (that is commonly involved in inducing cytokines) in all clinical isolates a similar finding that was also documented by [48] on samples collected from children admitted in Upstate Golisano Children's hospital, New York. Production of cytokines assist in the virulence of *Staphylococcus aureus* through modulation of the function of immune cells including neutrophils as well as triggering lysis of epithelial cells [9].

Further findings indicated that seven of the ten *Escherichia coli* isolates were carrying the cnf1 gene which helps the strain to escape from phagocytes as also documented by [50]. This assist this bacteria from being eliminated by the human body's phagocytic cells and the gene helps the bacteria to avoid this by provoking an increased adherence of polymorphonuclear leukocytes onto epithelial cells thus decreasing the rate of bacterial phagocytosis [51]. According to this study, the prevalence of magA and rmpA was reported to be 33% and 67% respectively and this was in agreement with the findings of [52] on molecular detection of capsular polysaccharide genes of *Klebsiella pneumoniae* on clinical samples collected from Hilla and Merjan Teaching hospital, Babylon. These genes therefore assist *K. pneumoniae* to produce capsules which have been found to protect them against phagocytic activities by the cells involved hence survival [53]. All *Pseudomonas aeruginosa* isolates in this study harbored lasL (600bp) and gyrB (222bp) a finding that is in tandem to the study findings on biopsy samples collected from Khatam-al-Anbia and Shahid Motahari hospitals, Iran by [54]. These genes assist *Pseudomonas aeruginosa* in quorum sensing that enhances their ability to form biofilms and produce other virulence traits like pyocyanin production which they commonly use to reduce ferric ions to ferrous state that is readily taken up as a nutritional requirement as well as reducing silver ions to the metallic state resulting in their lower concentration which is in consistent with its survival [55]. Contrary to these are the findings of [56] who detected 16srRNA, ToxA and exoS virulence factor genes from wound and burn swab samples collected from Al-Diwaneyah hospital, Iran. This differences could be attributed to the various environs where the samples were picked from for the analysis. Presence of these virulence traits genes is a worrying finding as they clearly demonstrate how pathogenic and virulent these genes are and this can have a negative effect in their management strategies.

The physicochemical and nutritive components in this study are comparatively similar to previous reports for stingless bees in Oromia, Ethiopia [57] but contrary to our findings, a lower moisture content value (25-27mg/g) was reported for Nigeria stingless bee honey [58], and higher moisture (199.0 – 419mg/g) and lower pH (3.15-4.66) from stingless honey samples in Brazil [59]. This moisture content differences are due to climate, season and moisture content of the original plant nectar variations since at different temperatures honey dehydrates very fast. High moisture content is one of the causes of quality deterioration but when maintained at low levels it protects skin maceration by keeping the injured tissues moist as well as preventing growth of bacteria and other microorganisms [60]. According to our findings, higher mean moisture content were were recorded

by honey samples from Kibimgor and Koriema compared to Mukutani and Maoi due to the differences in altitude levels. Minerals in honey protects the tissues against free radical damage and from our study, all minerals were present in detectable amounts across all honey collection regions with potassium being the most abundant element. This is a consistent finding by other authors who considered this mineral to be the most quantitatively important in honey followed by calcium, sodium, iron and magnesium [61]. This indicates the botanical and geographical origins of the honey samples as well as contributing in antibacterial activity through enhanced electrical conductivity [62]. Water soluble vitamins are immune system stimulators and confer antimicrobial activity [63]. Our findings did indicate the presence of trace water soluble vitamins amount with very little mean value detected from Maoi and no any detections made in Kibingor for Vitamin B5. This did differ with those of [64] who reported high values for vitB5 and VitB3 on Gelam and Acacia honey while other vitamins were not detected.

Phenols' antioxidant capacity result from the combined activity of flavonoids [65]. Our findings did indicate presence of low mean carotenoids, phenolics and flavonoids on samples collected from Maoi while the values increased spontaneously for samples collected from Koriema region. These findings concurred with those reported by [66] from their study on Stingless bee in the Southern Malaysia, but contrary to these findings, higher Total Phenolic Content values (196mgGAE/100G)) and lower Total Flavonoid Content (18.511mgRE/100g) has been reported by [63 & 67] respectively. Other findings of a study by [68] reported that Kelulut honey from East coast region showed highest values for phenolics (116.94 51.11mgGAE/100g), lower flavonoids (7.9 0.49mgQE/100g) and carotenoids (4.61 0.38mg/100g). Our findings indicated that the analyzed honey samples contain adequate phenolic and flavonoid compounds to cause inhibition of the growth of pathogenic bacteria by disturbing the function of the cell membrane. These diversity in concentrations could be due to the differences in the geographical floral origins, specific climatic conditions and the types of bee species [69].

Based on our findings it was deduced that Gram- positive isolates exhibited larger zones of inhibition compared to Gram-negative isolates. These could be attributed to the fact that Gram positive isolates have a larger peptidoglycan layer moiety on the cell membrane and hence they are easily broken down by various antibacterial agents as compared to Gram negative isolates [70]. This finding is in tandem to a study by [68] on the antibacterial activity of stingless bee honey collected from Amazonas State

against bacterial isolates. This could be attributed to the fact that stingless bee honey has been found to alter the outer membrane structure by changing the hydrophobic properties or mutations [55].

The MIC and MBC values in this study indicated that stingless bee honey samples have bactericidal and bacteriostatic activities potential against both control and wound isolates at reasonably low doses. Growth retardation and complete inhibition on all test organisms was observed at 62.5 to 250µg/ml with complete inhibition at very low concentration. This was similar to other studies conducted elsewhere [71, 72 & 73]. Contrary to our findings is a study conducted by [74] in Ethiopia indicating that the growth of *Escherichia coli* and *Staphylococcus aureus* were completely inhibited at 650µg/ml while *Pseudomonas aeruginosa* was inhibited at 750µg/ml. This could be due to variations in cellular organizations as well as the variations in honey composition. It is from the findings of this study, that we recommend that stingless bee multifloral honey be considered for approval in therapeutic use.

5.0 CONCLUSION

Millions of chronic wounds continue to be diagnosed globally every year with evidence of bacterial infections facilitating long hospital stays, high treatment costs and further delay of wound healing. This could be due to the polymicrobial environment associated with these wounds which creates a favorable environment for the exchange of resistance genes between microorganisms. The antibacterial, antioxidant and medicinal properties such as anti-inflammatory and anti-cancer of stingless bee honey are highly dependent on its composition which depends on the climatic, environmental conditions as well as the diversity of the nectar composition of each producing plant species. The composition of stingless bee honey in this study indicates significantly great nutritional, physicochemical, phytochemical, antioxidant parameters and antimicrobial properties.

6.0 ACKNOWLEDGEMENTS

1. William Kibyegon for his guidance through the vast Marigat Sub County.
2. Medical superintendent, County Referral Hospital, Nakuru for authorization of my access to the patients in the hospital.
3. Chief Medical Officer, Egerton University; Dr. G.K. Wahome for his authority the use of medical laboratory.

4. Laboratory technicians (Mr. Paul Kamau, Mr. Gerald Areba and Mr. Otieno), Biochemistry, Veterinary Sciences and Molecular Sciences laboratories, Egerton University
5. Mr. Robert Kimutai for his enormous input in data analysis.
6. My entire family for your emotional and psychological support during this endevour.

REFERENCES

1. Dinda V, Revathi G, Kariuki S, Hakean A. Pattern of pathogens and their sensitivity isolated from surgical site infections at the Aga Khan University Hospital, Nairobi, Kenya. *Ethiopian Journal of Health Sciences.* 2013; 23(2):141-149.
2. Amsellem P. Complications of reconstructive surgery in companion animals. *Vet Clin. Small Anim*. 2011; 41 (1):995-1006.
3. Negut I, Grumezescu V, Grumezescu MA. Treatment strategies for infected wounds. *Molecules.*2018; 23 (9):2392.
4. Tangcharoensathien V, Wibulpolprasert S, Nitayarampong S. Knowledge-based Changes to Health Systems: The Thai Experience in Policy Development. *Bulletin of the World Health Organization.* 2017; 82(10):750-756.
5. Pereira R F, Bartolo P J. Traditional Therapies for Skin Wound Healing, Mary Ann Liberty. *Inc. Publishers.* 2016; 5(5):208-229.
6. Mandal DM, Mandal S. Honey: Its medicinal property and antibacterial activity. *Asian Pac J Trop Biomed.* . 2011; 1(2):154-160.
7. Arawwawala M, Hewageegana S. Health Benefits and Traditional Uses of Honey: A Review. *Journal of Apitherapy.* 2017; 32(1):9.
8. Almasaudi S. The antibacterial activities of honey. *Saudi Journal of Biological Sciences*. 2021; 28(4):2188-2196.
9. Buba F Gidado A, Shugaba A. Physicochemical and microbiological properties of honey from North east Nigeria. *Biochem Anal Biochem*. 2013; 2(142): 61-67.
10. Abd jalil M, Kasmuri A, Hadi H. Stingless Bee Honey: The Natural Wound Healer. *Skin Pharmacol. Physiol.* 2017; 10(30):66-75.
11. Ezenwa L, Omondi P, Nwagbara M, Gradebo A, Bada, B. Climate variability and its effects on gender and coping strategies in Baringo County, Kenya. J. *Appl. Sci. Environ. Manage.* 2018; 22(5):699-706.

12. Mo ALF. Climate Risk Profile for Baringo County. Kenya County Climate Risk Profile Series. The Ministry of Agriculture, Livestock and Fisheries (MoALF), Nairobi, Kenya. 2017

13. Mohammed A, Endris Seid M, Gebrecherkos T, Tiruneh M, Moges F. Bacterial isolates and their antimicrobial susceptibility patterns of wound infections among inpatients and outpatients attending the University of Gondar Referral Hospital, Northern Ethiopia. *International Journal of Microbiology*. 2017:10.

14. Smith S. Determining Sample Size. How to ensure you get the correct sample size. *Qualtries LLC.* 2013; 4(1): 1-6.

15. Varier R. extracting Honey from our Stingless Bee Hive. https://.youtube.com. 2014.

16. Kothari CR. Research Methodology. *New Age International*. 2007; 18 (2):1-401.

17. Fox JD, Robyt S. Miniaturization of Three Carbohydrates Analysis Using a Micro-sample Plate Reader. *Journal of Food Quality*. 2018; 195 (8): 93-96.

18. Bogdanov S, Jurendia T, Sieber R, Gallmann P. Honey for nutrition and health: A review. *Journal of the American College on Nutrition*. 2009; 10 (4):745.

19. Reeb J, Milota M. (1999). Moisture Content by the Oven-dry Method for Industrial Testing. Western Dry Kiln Association. *Scientific Research*. 1999; 94(1): 57-90.

20. Major N, Makorvic K, Kripan M, Saric G, Hruskar M, Vahcic, N. Rapid Honey Characterization and Botanical Classification by an Electronic tongue. *Talanta.* 2011; 85 (16):569-574.

21. Kerkvliet D. Screening Methods for the Determination of Peroxide Accumulation in Honey and Relation with HMF Content. *Journal of Apicultural Research*. 2015; 35(3): 3-4.

22. Silva DJ, Queiroz AC. Determination of Total Nitrogen and Crude Protein in Portuguese. *Biological Methods*, 3rd Edition. 2002; 4(2):57-75.

23. Ferreira IC, Aires E, Barreira J, Estevinho LM. Antioxidant Activity of Portuguese Honey Samples: Different Contributions of the Entire Honey and Phenolic Extract. *Food Chemistry*. 2009; 114(4):1438-1443.

24. Ciulu M, Solinas S, Floris I, Panzanelli A, Pilo IM, Piu, CP, Spano N, Sanna G. RP –HPLC determination of water-soluble vitamins in honey. *Journal of homepage.* 2011; (2):924-929.

25. Pohl P. Determination of Metal Content in Honey by Atomic Absorption and Emission Spectrometries. *Trends Anal. Chem.* 2009; 28 (1):117-128.

26. Chaves M Flexas J, Pinheiro C. Photosynthesis Under Drought and Salt Stress. Regulation Mechanisms from Whole Plant to Cell. *Ann Bot*. 2008; 9(103):551-560.

27. Horwitz W.Official Method of Analysis of AOAC International. 17thEdition. *AOAC International*. 2000; 32(5):2-6

28. Socha R, Juszczak L, Pietrzyk S. Antioxidant Activity and Phenolic Composition of Herb-honeys. *Food Chemistry*. 2009; 113(2):568-574.

29. Isla M I, Craig A, Ordonez R, Zampini C, Sayago J, Bedascarrasbure E, Alvarez A, Salomon V, Maldonado L. Physico chemical and Bioactive Properties of Honey from Northwestern Argentina. *LWT-Food Science and Technology*. 2011; 44(9):1922-1930.

30. Thairu Y, Nasir I, Usman Y. Laboratory perspective of Gram staining and its significance in investigations of infectious diseases. *Sub-Saharan African Journal of Medicine.* 2014; 1(4):168-178.

31. Vesty A, Biswas K, Taylor W M, Gear K, Douglas G R. Evaluation of DNA Extraction Method on the Representation of Human Oral Bacterial and Fungal Communities. *PLOS ONE*.2017; 12(1):18.

32. Clermont O, Bonacorsi, S, Bingen E. Rapid and simple determination of *Escherichia coli* phylogenetic group. *Appl. Environ Microbiol*. 2000; 66(2):4555-4558.

33. Gautam V, Singhal L, Arora S, Jha C. Reliability of Kirby-Bauer Disk Diffusion Method for Detecting Carbepenem Resistance in Acinetobacter baumanii-calcoaceticus Complex Isolates. *Antimicrobial Agents and Chemotherapy*. 2013; 57(4):2003-2004

34. Wasihun GA, Kasa GB. Evaluation of antibacterial activity of honey against multidrug resistant bacteria in Ayder Referral and Teaching Hospital, Northern Ethiopia. *Springer*. 2016; 5: 842-845.

35. Mama M, Teshome T, Detomo J. Antibacterial activity of honey against *Methicillin-resistant Staphylococcus aureus*: A laboratory – based experimental study. *International Journal of Microbiology*. 2019, Article ID. 7686130:9.

36. Mullai V, Menon T. Bactericidal Activity of Different Types of Honey against Clinical and Environmental Isolates of *Pseudomonas aeruginosa*. *The journal of Alternative and Complementary Medicine.* 2007*;* 13(4):439-441.

37. Heuvelink AE, Van Den Biggelaar MA, Zwartkruis-Nahuis, MT. Occurrence of verocytotoxin-producing *Escherichia coli* 0571 on Dutch dairy farms. Journal of Clinical Microbiology. 36(12):3480-3487.

38. Codex Alimentarius Commission on Sugars, Codex Standard 12, Revised Codex Standard for Honey. *Standards and Methods*. 2001; 11(1):1-7.

39. Codex Alimentarius Commission. Food and Agriculture Organization of the United Nations. World Health Organization. 1999; 3:1-34.

40. Armstrong DG, Duerden BI, Bowler PG. Wound Microbiology and Associated Approaches to Wound Management. *Clin Microbiol Rev.* 2001; 14(2(:244-269.

41. Garba I, Lusa YH, Bawa, E. Antibiotics Susceptibility Pattern of *Pseudomonas aeruginosa* from Wounds in Patients attending Ahmachu Bello University Teaching Hospital, Zaria, Nigeria. *Nigerian Journal of Basic and Applied Sciences*. 2012; 20 (1):32-34.

42. Gumbart J, Beeby M, Jensen G, Roux B. *Escherichia coli* peptidoglycan structure and mechanics as predicted by atomic-scale simulations. *PLOS Compt. Biol.* 2014; 10(2):475.

43. Sultana S, Mawla N, Kawser S, Akhtar N. Current Microbial Isolates from Wound Swabs and Their Susceptibility Pattern in a Private Medical College Hospital in Dhaka City. *Delta Medical College Journal*. 2015; 3(1):25-27.

44. Prabna S P Rani D M, Lakshorni K, Chitralekha S. Study of bacteriological etiology of wound infection. *Research Journal of Pharmaceutical, Biological and Chemical Sciences*.2015; 6(2):270-274.

45. Rai S, Yadar U, Dutt Pant N, Yakha J, Tripathi P, Paudel A, Lekhak B. Bacteriological Profile and Antimicrobial Susceptibility Patterns of Bacteria Isolated from Pus /wound Swabs from Children Attending a Tertiary Care Hospital in Kathmandu, Nepal. *International Journal of Microbiology*. 2017; 17(2):2-3.

46. Bowler GP, Duerden IB, Armstrong GD. Wound Microbiology and Assorted Approaches to Wound Management. *Clin Microbiol / Rev*. 2001; 14(2):244-269.

47. Tacconelli E, Carrara E, Salvoldi A, Harbarth S, Mendelson M, Monnet D L, Magrini, N. Discovery, research, and development of new antibiotics: The WHO priority list of antibiotic-resistant bacteria and tuberculosis. *The Lancet Infectious Diseases*. 2017; 18(04): 318-327

48. Suryadevara M, Clark EA, Wolk MD, Carman A, Rosenbaum FP, Shaw J. Molecular Characterization of Invasive Staphylococcus aureus Infection in Central New York Children: Importance of Two Clonal Groups and Inconsistent Presence of Selected Virulence Determinants. *Journal of Pediatric Infectious Diseases Society.* 2013; 2(1):30-39.

49. Becker S M B, Frankel O, Schneewind, Missiakas, D. Release of protein from the cell wall of *Staphylococcus aureus*. *Proceedings of the National Academy of Sciences of the United States of America*. 2014; 111(04):1574-1579.

50. Petkoisek Z, Elersic K, Gubina M, Zgur-Bertok D, Starcic E, M. Virulence Potential of *Escherichia coli* Isolates from Skin and Soft tissue Infections. *J Clin Microbiol.* 2009; 47(2):1811-1817.

51. Chakraborty S, Kaur S, Guha S, Batra K S. "The multifaceted roles of neutrophil gelatinase associated lipocalin (NGAL) in inflammation and cancer". *Biochimica et Biophysica Acta-Reviews on cancer*. 2012; 1826(01):129-169.

52. Al-Khafaji J. Genotyping and Detection of Some Virulence Genes of *Klebsiella pneumoniae* Isolated from Clinical Isolates. *Medical Journal of Babylon*. 2013; 10 (2):387-399.

53. Amraie H, Shakib P, Rouhi S, Bakhshandeh N, Zamanzad B. prevalence assessment of magA gene and antibacterial susceptibility of Klebsiella pneumonia isolated from clinical specimens in Shahrekord, Iran. *Iran J. Microbiol*. 2014; 6(05):311-316.

54. Aghamollaei H, Maghaddam M, Koashki H, Heisat M, Mirnejad R, Barzi N. Detection of *Pseudomonas aeruginosa* by a Triplex Polymerase Chain Reaction Assay Base on laslR and gyrB Genes. *Journal of Infection and Public Health.* 2015; 8(4):314-322.

55. Lee J. & Zhang L. The hierarchy quorum sensing network in Pseudomonas aeruginosa. *Protein Cell*. 2015; 6 (4): 26-41.

56. Shaebth L J. Molecular Identification and Sequencing of *Pseudomonas aeruginosa* Virulence Genes Among Different Isolates in Al-Diwaneyah Hospital. *Iraqi Journal of Vetrinary Sciences*. 2018; 32(2):183-188.

57. Gela A, Hora A, Z, Kebebe D, Gebresilassie A. Physicho-chemical Characteristics of Honey Produced by Stingless bees (*Meliponula becarii*) from West show Zone of Oromania Region, Ethiopia. *National Center for Biotechnology Information*. 2021: 7(4):10-11.

58. Onyenso AL, Akachuku CO. Physico -chemical Properties of Honey Produced by Two Stingless bee Species *Trigonacarbonaria* and *Melipona beecheii* in South-Eastern Nigeria. *J. Agric Forest Soc Sci.* 2011; 9(1):19-26.

59. Bruno AS, David R, Ortruol M B, Tim Heard B. Composition of Stingless bee Honey: Setting Quality Standards. *Interciencia.* 2006; 31(2):867-874.

60. Almasaudi BS. The Antibacterial Activities of Honey. *Saudi Journal of Biological Science*. 2020; 28(2):4-6.

61. Biluca CF, Braghini F, Gonzaya L, Oliveira AC, Fett R. Physicochemical Profiles, Minerals & Bioactive Compounds of Stingless bee Honey (*Meliponinae). Journal of Food Composition and Analysis*.2017; 50 (1):61-69.

62. Aazza, S, Lyoussi, B, Antunes D, Miguel GM. Physicochemical characterization & antioxidant activity of commercial honeys. *Journal of Food Science*. 2013; 78(8): 78(8):C1159-C1165.

63. Majtan J, Sojka M, Palenikova H, Bucekova M, Majtan V. Vitamin C enhances Antibacterial Activity of Honey against Planktonic and Biofilm –Embedded Bacteria. *Molecules*. 2020; 25(4):992.

64. Chua LS, Adnan NA, Abdul-Rahaman N L, Sarmidi MR. Effect of Thermal Treatment on the Biochemical Composition of Tropical Honey Samples. *International Food Research Journal*. 2014; 21 (2): 773-774.

65. Sagdic O, Silici S, Ekici L. Evaluation of the Phenolic Content, Antiradical, Antioxidant, and Antimicrobial Activity of Differrent Floral Sources of Honey. *International Journal of Food Properties*.2013; 16 (3):658-666.

66. Bakar A F, Sanusi BS, Bakar AI, Congo JO, Mian Z. Physicochemical and Antioxidant Potential of Raw Unprocessed Honey from Malaysian Stingless bee. *Pakistan Journal of Nutrition.* 2017; 16(11):888-894.

67. Menda A, Lamien CE, Romito M, Milogo J, Nacoulmaand O, G. Determination of the Total Phenolic, Flavonoid and Proline Contents in Burkina Fasan Honey, as well as their Radical Scavenging Activity. *Food Chemistry.* 2005; 91(3):571-577.

68. Keng BC, Haron HM, Talib, AR, Subramaniam P. Physical Properties, Antioxidant Content and Anti-oxidation Activities of Malaysian Stingless Kelulut (Trigona spp.) Honey. *Journal of Agricultural Sciences.* 2017; 9(13):32.

69. Braide W, Oranusi SU, Akaluka CK, Nwaoguikpe RN, Akobundal CI, Peter NI. Antibacterial efficacy of crude and diluted honey on four wound isolates. *J Microbiol*. 2012; 1(1):1-4.

70. Exner M, Bhattacharya S, Christiansen B, Gebel J, Goroncy-Bermes P, Hartmann P, Heeg P, Kramer A, Larson E. Antibiotic resistance: what is so special about multi-drug resistant Gram – negative bacteria? *GMS Hyg. Infect. Control*. 2017; 12(2): 413-415.

71. Chan-Rodriguez DM, Raman-Sierra J, Lope-Ayora J, Sauri-Duch E, Ceuvas- Gilory L, Ortiz-Vazquez E. Antibacterial Properties of Honey Produced by *Melipona beiaecheii* and

Apis mellifera Against Food born Microorganisms. *Food Science Biotechnology*. 2012; 21(3):905-909.

72. Ewnetu Y, Lemma W, Birhane N. Antibacterial Effects of Apis mellifera and Stingless bees Honey on Susceptible and Resistant Strains of *Escherichia coli*, *Staphylococcus aureus* and *Klebsiella pneumoniae* in Godar, Norwest Ethiopia. *BMC Complementary and Alternative Medicine.* 2013; 13 (1):269.

73. Getaneh A, Belyhun Y, Moges F, Anagaw B, Destaw B, Unakal C. In vitro Assessment of the Antimicrobial Effect of Ethiopian Multi-floral Honey on Methicillin Resistant *Staphylococcus aureus*. *J Curr Res Rev.* 2013; 5(11):64-72.

74. Ahmed M, Sahile S, Subramanian C. Evaluation of Antibacterial Potential of Honey Against Some Common Human Pathogens in North Gordon Zone of Ethiopia. *Int J Pure Appl Zool. 2014;* 2 (4): 286-295.

Click here to access/download;Figure;FIGURE LEGEND [STINGLESS III].docx

FIGURE LEGENDS

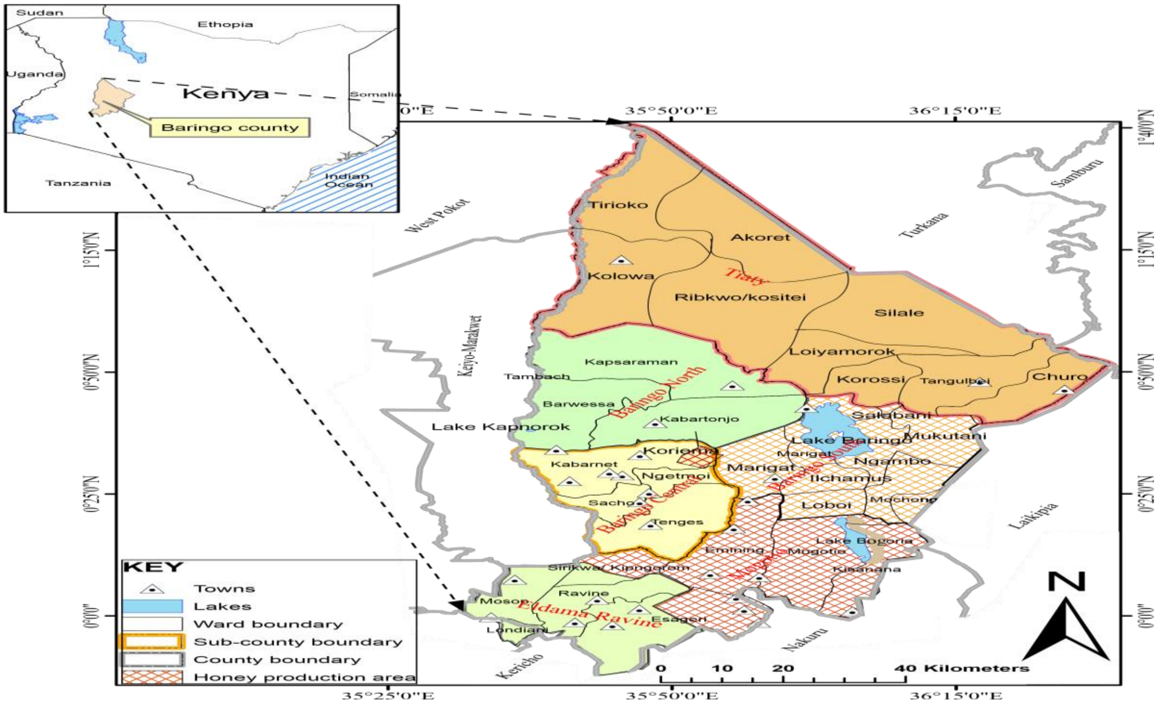

Figure 1: Geographical origin of honey samples

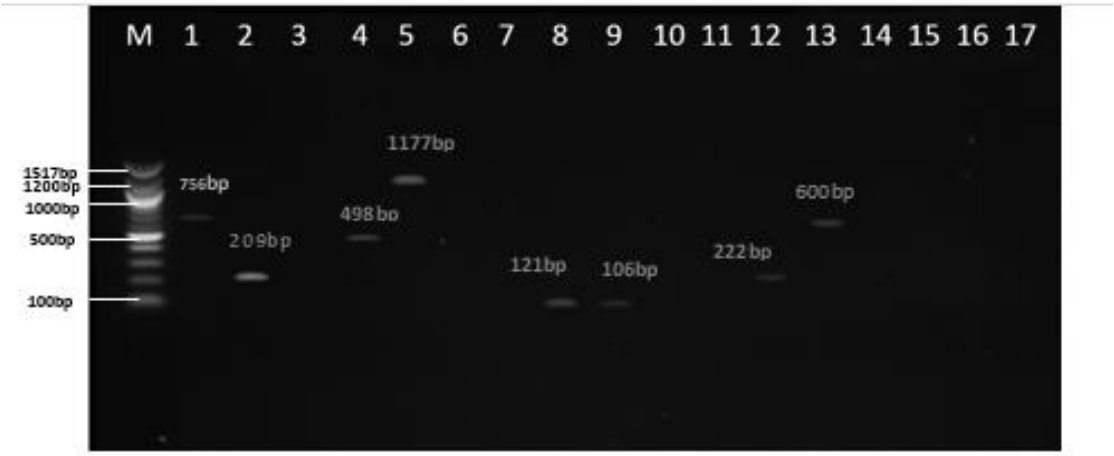

FIGURE 2: Agarose gel (1%) electrophoresis showing the typical amplicons of the virulence genes products of the bacterial isolates *Staphylococcus aureus* (Lane 1 and 2), *Escherichia coli* (lane 4 and 5), *Klebsiella pneumoniae* (Lane 8 and 9) and *Pseudomonas aeruginosa* (Lane 12 and 13). Lanes 6 and 10 were negative for the genes and hence did not indicate any amplicons while lanes 3, 7 and 11 were set as negative control. M is a ladder (standard) containing a mixture of DNA fragments of predetermined sizes that can be compared against the unknown DNA samples.

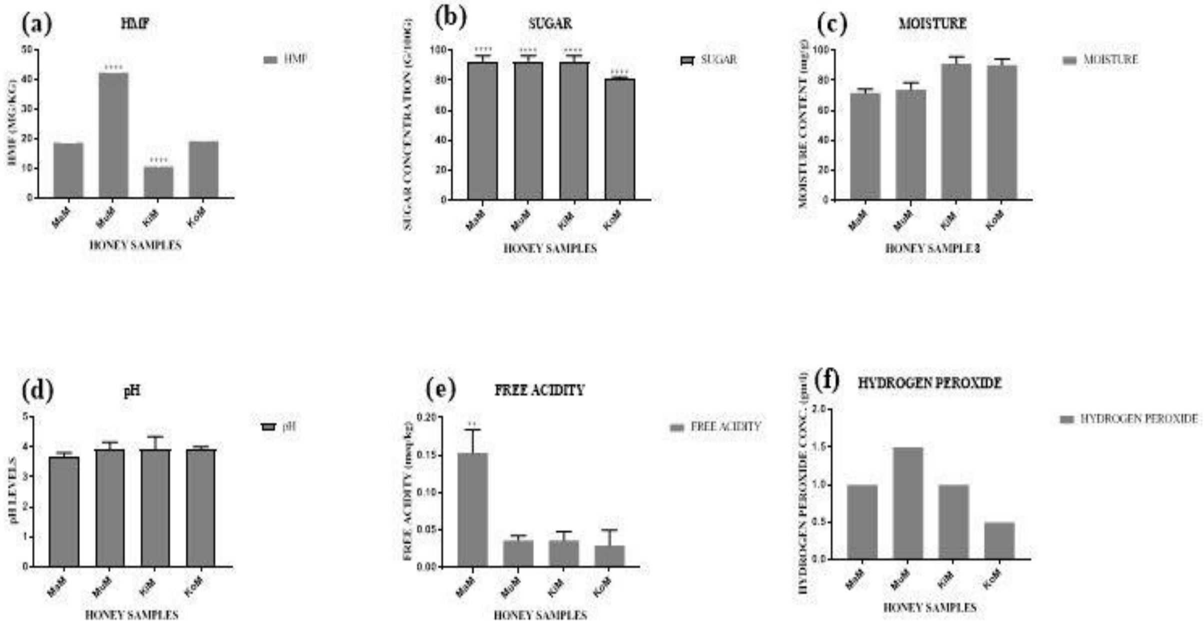

Figure 3: Physicochemical and nutritive properties of stingless bee honey; Hydromethylfurfural – HMF (a), Sugar (b), Moisture (c), pH (d), Free acidity (e) and Hydrogen peroxide (f). The values are represented in mean ± SD as error bars represent Standard deviation (SD). Significant values ($P<0.05$) are represented by stars on the bars (*$P< 0.05$, **$P<0.01$, ***$P<0.001$ and **** $P<0.0001$). (**KEY**: **MaM** – Maoi Meliponin, **MuM** – Mukutani Meliponin, **KiM** – Kibigor Meliponin, **KoM-** Koriema Meliponin,).

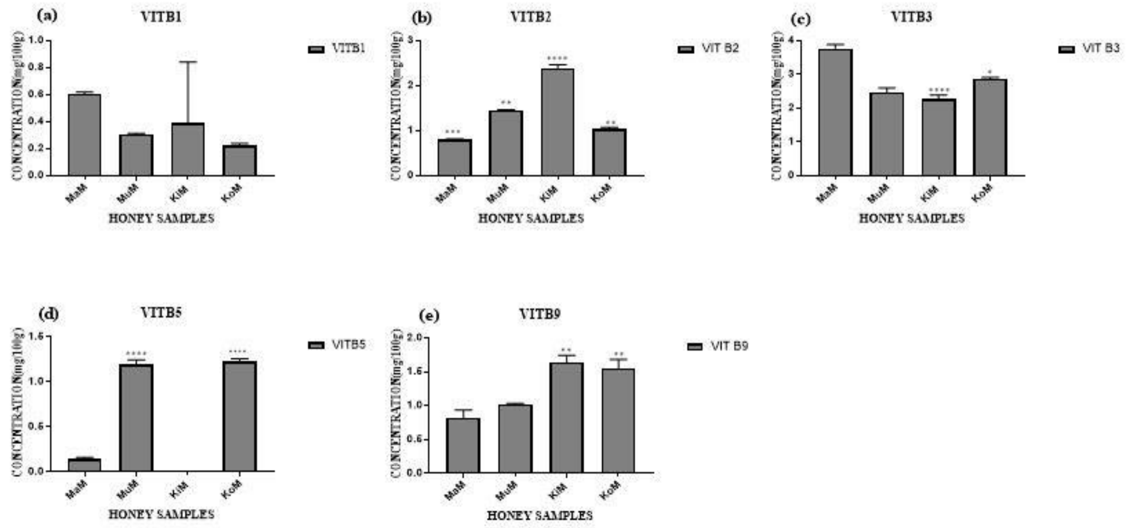

Figure 4: Physicochemical and nutritive properties of stingless bee honey; Vitamin B_1 (A), Vitamin B_2 (B), Vitamin B_3 (C), Vitamin B_5 (D) and Vitamin B_9 (E). The values are represented in mean ± SD as error bars represent Standard deviation (SD). Significant values ($P<0.05$) are represented by stars on the bars (*$P< 0.05$, **$P<0.01$, ***$P<0.001$ and **** $P<0.0001$) (**KEY**: **MaM** – Maoi Meliponin, **MuM** – Mukutani Meliponin, **KiM** – Kibigor Meliponin, **KoM** - Koriema Meliponin,).

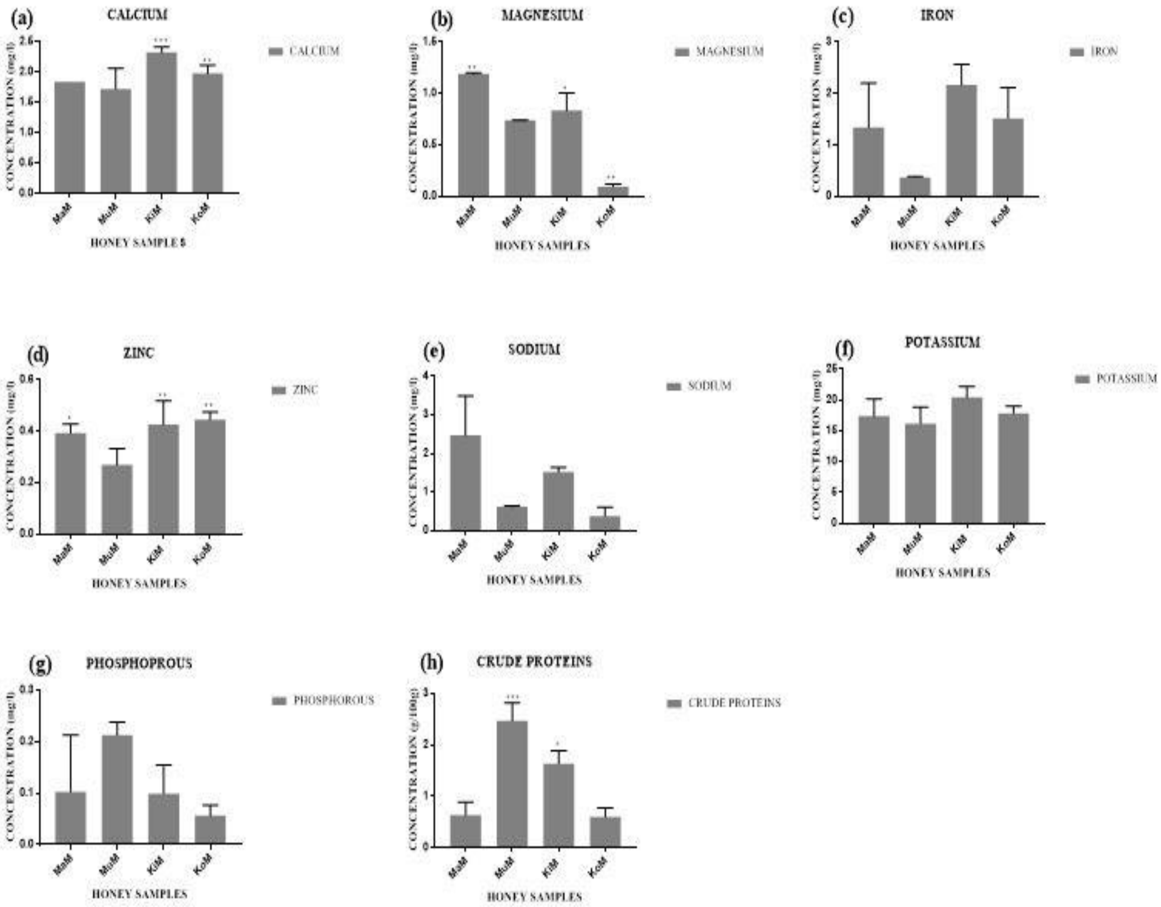

Figure 5: Physicochemical and nutritive properties of stingless bee honey; Calcium – ca (a), Magnesium – mg (b), Iron- fe (c), Zinc –zn (d) Sodium – Na (e), Potassium - K (f), Phosphorous - P (g) and Crude proteins (h). The values are represented in mean ± SD as error bars represent Standard deviation (SD). Significant values (P<0.05) are represented by stars on the bars (*P< 0.05, **P<0.01, ***P<0.001 and **** P<0.0001) (**KEY: MaM** – Maoi Meliponin, **MuM** – Mukutani Meliponin, **KiM** – Kibigor Meliponin, **KoM-** Koriema Meliponin).

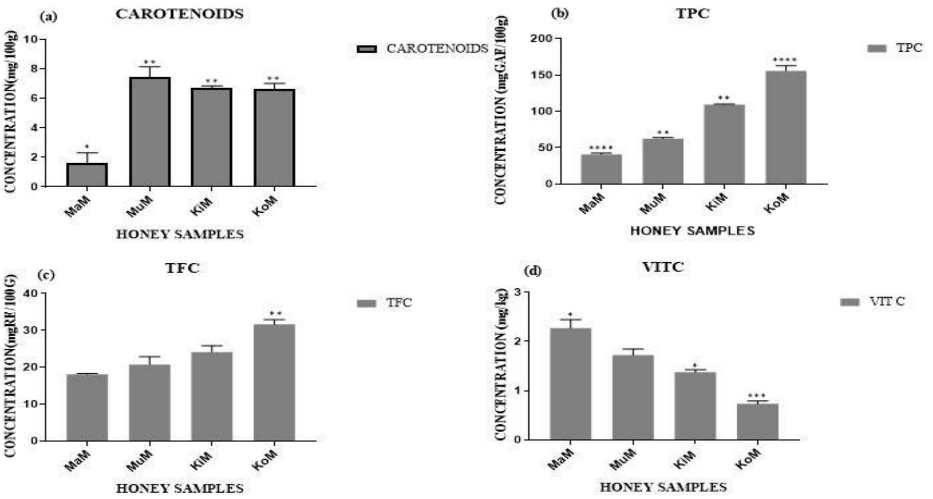

Figure 6: Phytochemical properties of stingless bee honey; Total flavonoid content – TFC (a), Total phenolic content – TPC (b), Carotenoids (c) and Vitamin C (d). The values are represented in mean ± SD as error bars represent Standard deviation (SD). Significant values ($P<0.05$) are represented by stars on the bars (*$P< 0.05$, **$P<0.01$, ***$P<0.001$ and **** $P<0.0001$) (**KEY: MaM** – Maoi Meliponin, **MuM** – Mukutani Meliponin, **KiM** – Kibigor Meliponin, **KoM**- Koriema Meliponin).

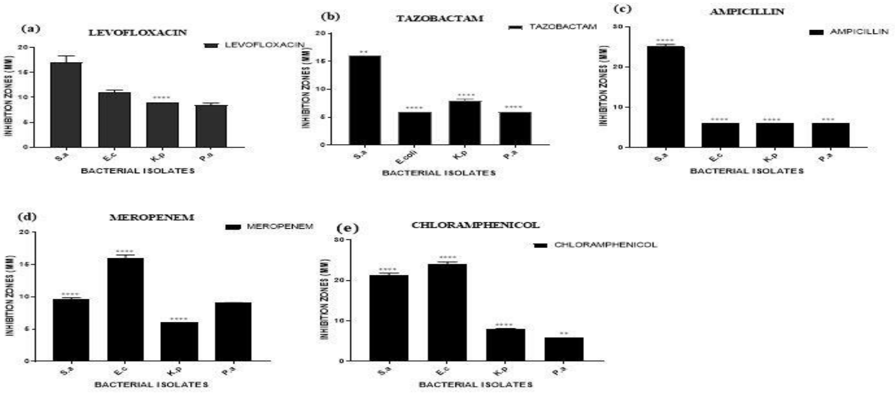

Figure 7: Antibacterial activity of commonly used antibiotics against bacterial isolates from burns and cutaneous wounds. (a) Levofloxacin (5 µg), (b) Tazobactam (110 µg), (c) Ampicillin (10 µg), (d) Meropenem (10 µg), (e) Gentamicin (10 µg) and (f) Chloramphenicol (30 µg). The values are represented in mean ± SD as error bars represent Standard deviation (SD). Significant values ($P<0.05$) are represented by stars on the bars (*$P< 0.05$, **$P<0.01$, ***$P<0.001$ and **** $P<0.0001$. (KEY: S. a- *Staphylococcus aureus*, E. c. - *Escherichia coli*, K. p. - *Klebsiella pneumoniae* and P.a. - *Pseudomonas aeruginosa*).

Click here to access/download;Figure;TABLE LENGENDS [stingless II].docx

TABLE LENGENDS [STINGLESS BEE HONEY]

Table 1: Cultural proportion of pure bacterial isolates from infected cutaneous wounds.

Type of swabs	No. collected	No. infected	No. of pure isolates	Frequency (%)
Burn	6	4	6	14.7
Wound	28	24	37	82.4
**Total**	34	28	43	97.1

Table 2: Proportion of Gram staining reactions of bacterial isolates from the collected wound swabs

Bacterial isolates	Gram reaction	Morphology	Swabs	Frequency (%)
Gram stain 1	Gram Positive	Cocci	10	29.4
Gram stain 2	Gram Negative	Rods (Single/pair)	12	35.3
Gram stain 3	Gram Negative	Rods (Plump/short chains	4	11.8
Gram stain 4	Gram Negative	Rods (Slimmer)	3	8
Gram stain 5	Nil		5	14.7
**Total**			34	91.2

Table 3: Biochemical tests

		Gram reaction			
**Biochemical test**		**Gram-negative n=3 (75%)**		**Gram -positive n=1(25%)**	
Coagulase				+ (35%)	- 0%)
TSI	Butt/slope	Acid/Acid	Acid/ Acid	Alkaline/Alkaline	Alkaline/ Alkaline
		+ (37.2%)	+	- (62.8%)	-
	Gas	+ (37.2%)	+	- (62.8%)	-
	H2S	-	-	-	-
MethylRed		+ (13.95%)	- (23.26%)		
Oxidase		-	-	+ (27.91%)	- (13.95%)
Indole					
Voges prosker		NT	NT	NT	NT
Citrate		NT	NT	NT	NT
Urease		NT	NT	NT	NT
Possible pathogen		*E.coli* (13.95%)	*K. pneumoniae* (23.26%)	*P. aeruginosa* (27.91%)	*S. aureus* (35%)

Table 4: Mean inhibition of the stingless bee honey dilutions (10, 20, 50 and 75 x 10^4 µg/disc) against the bacterial isolates

		Stingless honey samples (*Meliponin*) zones of inhibition (mm)			
**Isolates**	**Concentration(x10^4µg/disc)**	MuM	MaM	KiM	KoM
S. aureus	10	6.4 ± 0.00	6.1 ± 0.00	6.0 ± 0.00	6.0 ± 0.00
	20	6.4 ± 0.00	6.2 ± 0.00	6.0 ± 0.00	6.0 ± 0.00
	50	12.8 ± 2.17	20.08 ± 0.67	14.62 ± 0.33	21.73 ± 0.37
	75	15.29 ± 0.31	22.9 ± 0.00	17.56 ± 0.53	25.22 ± 0.49
E. coli	10	6.2 ± 0.00	6.3 ± 0.00	6.0 ± 0.00	6.1 ± 0.00
	20	6.4 ± 0.00	6.1 ± 0.00	6.0 ± 0.00	6.2 ± 0.00
	50	9.00 ± 0.37	7.11 ± 0.19	12.55 ± 0.22	7.59 ± 0.19
	75	12.48 ± 0.52	10.89 ± 0.00	15.87 ± 0.37	10.48 ± 0.00
K. Pneumoniae	10	6.1 ± 00	6.3 ± 0.00	6.0 ± 0.00	6.2 ± 0.00
	20	6.1 ± 0.00	6.0 ± 0.00	6.0 ± 0.00	6.6 ± 0.00
	50	13.48 ± 0.27	19.65 ± 0.38	15.43± 0.46	14.88 ± 0.22
	75	18.67 ± 0.39	22.96 ± 0.00	18.86 ± 0.69	17.14 ± 0.16
P. aeruginosa	10	6.3 ± 0.00	6.0 ± 0.00	6.0 ± 0.00	6.0 ± 0.00
	20	6.3 ± 0.00	6.1 ± 0.00	6.0 ± 0.00	6.6 ± 0.00
	50	16.28 ± 0.06	15.45 ± 0.35	17.89 ± 0.03	13.52 ± 0.41
	75	19.75 ± 0.12	17.19 ± 0.03	21.63 ± 0.08	18.48 ± 0.35

Table 5: Average inhibition of the stingless honey samples (10x10^4, 20x10^4, 50x10^4 and 75x10^4 µg/ml) and control wound bacterial isolates

**Isolates/ PC**	**Stingless bee samples (*Meliponin*) inhibition zones (mm)**
S. aureus	18.79 ± 4.49
ATCC 25923 *(S.aureus)*	16.21 ± 2.64
E. coli	10.76 ± 2.46
ATCC 25922 *(E.coli)*	11.00 ± 2.44
K. pneumonia	17.55 ± 2.48
ATCC 27736(*K.pneumoniae*)	17.00 ± 2.45
P. aeroginosa	18.81 ± 1.24
ATCC 27853(*P.aeruginosa*)	17.85 ± 1.28

Table 6: Minimum bacteriostatic and bactericidal concentration – MIC/MBC (µg/ml) of the bacterial isolates subjected to different concentrations of stingless bee honey samples (500, 250, 125, 62.5, and 31.25µg/ml).

	Staphylococcus aureus		***Escherichia coli***		***Pseudomonas aeruginosa***		***Klebsiella pneumoniae***	
	MIC µg/ml	MBC µg/ml	MIC µg/ml	MBC µg/ml	MIC µg/ml	MBC µg/ml	MIC µg/ml	MBC µg/ml
**MuM**	62.5	250	250	125	62.5	125	125	125
**MaM**	125	250	250	250	125	125	62.5	62.5
**KiM**	125	125	125	125	62.5	62.5	62.5	62.5
**KoM**	62.5	125	125	250	62.5	125	62.5	125

KEY: **MIC** – Minimum inhibition concentration, **MBC** – Minimum Bactericidal concentration. **MaM** – Maoi Meliponin, **MuM** – Mukutani Meliponin, **KiM** – Kibigor Meliponin, **KoM**- Koriema Meliponin).

Click here to access/download
Supporting Information
Supplementary data (Stingless II).docx
