# Supplementary material for: Stingless bee honey: Nutritional, physicochemical, phytochemical and antibacterial validation properties against wound bacterial isolates
Source: PLoS One. 2024 May 14;19(5):e0301201. doi: 10.1371/journal.pone.0301201 (PMC11093306; doi:10.1371/journal.pone.0301201)
Supplement: Attachment — Submitted filename: RESPONSE TO REVIEWERS.docx [file pone.0301201.s015.docx]

Dear to reviewers,

We thank you for the time and effort you have put into evaluating our manuscript “*Stingless bee honey: Nutritional, Physicochemical, Phytochemical and antibacterial validation properties against wound bacterial isolates.’’* (Manuscript Number: PONE-D-22-28752).

We have provided the original and uncropped image of gel data image in the “Supporting image” document.

Thank you again for your thoughtful reviews, looking forward to hearing from you soon.

Sincerely,

Miriam Wanjiru Mwangi
