# Supplementary material for: Stingless bee honey: Nutritional, physicochemical, phytochemical and antibacterial validation properties against wound bacterial isolates
Source: PLoS One. 2024 May 14;19(5):e0301201. doi: 10.1371/journal.pone.0301201 (PMC11093306; doi:10.1371/journal.pone.0301201)
Supplement: Attachment — Submitted filename: Response to Reviewers.docx [file pone.0301201.s016.docx]

Dear Reviewers,

We thank you for the time and effort you have put into evaluating our manuscript "Stingless bee honey: Nutritional, physicochemical, phytochemical and antibacterial validation properties against wound bacterial isolates." (Manuscript Number: PONE-D-22-28752). We appreciate the thoughtful comments and suggestions provided by the reviewers. We have made several revisions to address reviewers’ concerns as indicated in Table 1.

Table1: Revisions guided by the reviewers comments

| Comments | Correction | Page |
| --- | --- | --- |
| Meliponines is not a stingless bee genus. Therefore Meliponines spp. does not exist. | Meliponines was deleted in the sentence | Page 1(Abstract)  Fourteenth sentence. |
| “Resin” replace by cerumen | Resin was replaced with cerumen | Page 1(Abstract)  Fifteenth sentence. |
| “Sacking” replace by sucking | Sacking was replaced by sucking | Page 4 subheading 2.2.2 Honey Sampling Techniques paragraph one 3^rd^ sentence. |
| “and” replace by, and ADD COMMA | Comma was added before the word and to read “, and” | Page 4 subheading 2.2.2 Honey Sampling Techniques paragraph one 2^nd^ sentence. |
| “[18]” are you sure this is a reference on analytical methods? | The correction was made by deleting “[18]” to read “Analysis was done as shown below” since “[18]” was a not reference on analytical methods | Page 4 subheading 2.3 Analysis of the physicochemical properties of the honey samples first sentence. |
| “2.0” replace by Two grams | The correction was made by replacing “2.0” with “Two grams” | Page 4 Subheading ***2.3.1 Determination of sugar content*** first sentence. |
| replace by o CAPITAL O | The correction was made by replacing o CAPITAL O to read (H_2_SO_4_). | Page 4 subheading ***2.3.1 Determination of sugar content*** paragraph one fourth sentence |
| c was highlighted | The correction was made by replacing c with capital C to read 70°C | Page 4 subheading ***2.3.2 Determination of moisture in honey samples*** |
| “titratable” replace by free | The correction was made by “titratable” replace by free to read “Determination of free acidity in honey samples”  In the first sentence the correction was made by replacing “titratable” by free to read  “The free acidity…” | Page 5 subheading ***2.3.4 Determination of free acidity in honey samples*** and the first sentence in the first paragraph |
| “10g” replace by ten grams | The correction was made by replacing “10g” by Ten grams | Page 5 subheading ***2.3.4 Determination of free acidity in honey samples*** and the second sentence in the first paragraph |
| “was” replace by were | The correction was made by replacing “was” by were to read “honey were …” | Page 5 subheading ***2.3.4 Determination of free acidity in honey samples*** the second sentence in the first paragraph |
| “10ml of 0.05M NaOH was added into the solution and back titrated to pH 8.3 using 0.05M HCL from a second burette and recorded.” deleted | The correction was made by deleting the sentence | Page 5 subheading ***2.3.4 Determination of free acidity in honey samples*** the fourth sentence in the first paragraph |
| “a blank is not needed to measure free acidity, better inform units of free acidity” | The correction was made by deleting the sentence “A blank determination procedure of 75 ml of distilled water was also run to pH 8.5, the free acidity is the acidity titrable with sodium hydroxide up to the equivalent point.” | Page 5 subheading ***2.3.4 Determination of free acidity in honey samples*** the last sentence in the first paragraph |
| replace by “hydrxymethylfurfural” | The correction was made by replacing “Hydroxy methyl furfural (HMF)” by “hydrxymethylfurfural” | Page 5 subheading ***2.3.5 Determination of hydrxymethylfurfural*** |
| “5g” replace by five grams | The correction was made by replacing “5g” by Five grams | Page 5 subheading ***2.3.5 Determination of hydrxymethylfurfural*** second sentence in the first paragraph |
| “was” replace by were | The correction was made by replacing “was” by were to read “honey were weighed...” | Page 5 subheading ***2.3.5 Determination of hydrxymethylfurfural*** second sentence in the first paragraph |
| Colour replace by color | The correction was made by replacing “colour” by color. | Page 6 subheading ***2.3.6 Determination of hydrogen peroxide in honey*** fourth sentence in the first paragraph |
| “Nutritive” replace by Nutritional | The correction was made by replacing “Nutritive” by Nutritional. | Page 6 subheading ***2.4 Nutritive Analysis of Honey*** |
| replace by o CAPITAL O | The correction was made by replacing o CAPITAL O to read (H_2_SO_4_), | Page 6 subheading ***2.4.1 Determination of sugar content*** fourth sentence in the first paragraph |
| colour.50 ml | The correction was made by adding space between color and.50 ml | Page 6 subheading ***2.4.2 Determination of Honey Protein Concentration*** eight sentence paragraph one. |
| HCL replace by LOWER CASE l | The correction was made by replacing L by a lower-case l to read HCl | Page 6 subheading ***2.4.2 Determination of Honey Protein Concentration*** nineth sentence paragraph one. |
| “folin-ciocalteu” replace by Folin-Ciocalteau | The correction was made by replacing “folin-ciocalteu” by Folin-Ciocalteu | Page 8 subheading ***2.5.1 Total Content of Phenolic Compounds (TCPC)*** second sentence paragraph one |
| “was” replace by  were | The correction was made by replacing “was” by were to read “Flavonoids were determined…” | Page 8 subheading ***2.5.1 Total Content of Phenolic Compounds (TCPC)*** first sentence paragraph two. |
| “were were” DELETE | The correction was made by deleting one of the repeated “were” in the sentence to read “…moisture content were recorded...” | Page 18 heading **4.0 DISCUSSION** nineth sentence paragraph four. |
| The authors failed to make the corrections described in the previous review, regarding the standardization of measurement units (mass, temperature), mainly in materials & methods, lacking space between the value and the measurement unit. Please refer to the previously submitted detailed review and make corrections. | The corrections were made by standardizing the measurement units for mass and temperature, mainly in the materials & methods section, and ensured there is a space between the value and the measurement unit. | Materials & methods section (Page 5 to 27) |

We appreciate the opportunity to revise our manuscript and believe that the changes we have made have significantly improved the quality and clarity of our work. We hope that these revisions have addressed the reviewers' concerns and that our manuscript will be considered for publication in PLOS ONE.

Sincerely,

Miriam Wanjiru Mwangi
